# Housing Displacement, Employment Disruption, and Mental Health After the 2023 Maui Wildfires

**DOI:** 10.1001/jamapsychiatry.2026.0044

**Published:** 2026-03-11

**Authors:** Ruben Juarez, Binh Le, Christopher Knightsbridge, Marsha Lowery, Alika K. Maunakea

**Affiliations:** 1 University of Hawai‘i Economic Research Organization, University of Hawai‘i at Manoa, Honolulu; 2 Department of Economics, University of Hawai‘i at Manoa, Honolulu; 3 Maui Wildfire Exposure Study, University of Hawai‘i, Honolulu; 4 Department of Anatomy, Biochemistry and Physiology, John A. Burns School of Medicine, University of Hawai‘i at Manoa, Honolulu

## Abstract

**Importance:**

Climate-related disasters are escalating in frequency and severity, yet their population-level mental health impacts—especially in racially and ethnically diverse and geographically isolated settings—remain poorly characterized. The 2023 Maui wildfires, one of the deadliest US wildfires in more than a century, offer a critical opportunity to quantify these effects.

**Objective:**

To examine associations between wildfire exposure and symptoms of depression, anxiety, and suicidal ideation and to assess indirect pathways of these associations through housing displacement and income disruption.

**Design, Setting, and Participants:**

This multiarm, propensity-weighted cross-sectional study compared adults residing within burn zones and outside burn zones on Maui and unexposed residents from other Hawaiʻi counties. The analytic sample included wildfire-exposed and unexposed adults enrolled between January 2024 and February 2025 through the Maui Wildfire Exposure Study and the UHERO Rapid Health Survey. Residential addresses at the time of the fires were geocoded and linked to official burn zone perimeters to determine exposure status. Data were analyzed from May to September 2025.

**Main Outcomes and Measures:**

The primary outcomes were self-reported depression (Center for Epidemiologic Studies Depression Scale), anxiety (7-item Generalized Anxiety Disorder scale), and suicidal ideation within the past 30 days, assessed using validated screening instruments.

**Results:**

The analytic sample included 2453 adults (1535 wildfire exposed and 918 unexposed), among whom mean (SD) age was 50.8 (16.3) years and 1502 participants (61.2%) were women. Wildfire exposure was associated with higher risk of depression (risk ratio [RR], 1.53; 95% CI, 1.20-1.94) and anxiety (RR, 1.67; 95% CI, 1.14-2.45) compared with unexposed individuals. Although suicidal ideation was more frequent among burn zone residents (RR, 2.15; 95% CI, 0.72-6.44), this association was not statistically significant. Residents outside burn zones showed significantly higher risk of suicidal ideation (RR, 2.65; 95% CI, 1.21-5.77). Mediation analyses indicated that housing displacement and income loss jointly accounted for more than half of the associations with depression and anxiety.

**Conclusions and Relevance:**

In this cross-sectional study, wildfire exposure and its socioeconomic consequences were associated with graded increases in psychological distress extending beyond the burn zone. These findings highlight the importance of integrating mental health care, housing stability, and economic recovery as central pillars of disaster response and climate resilience strategies.

## Key Points

**Question:**

What are the mental health consequences of wildfire exposure, housing displacement, and income disruption following the 2023 Maui wildfires?

**Findings:**

In this cross-sectional study of 2453 adults, residents within wildfire burn zones had a 53% higher risk of depression and 67% higher risk of anxiety compared with unexposed individuals, with suicidal ideation not significantly higher within burn zones but elevated among residents outside burn zones. Housing displacement and income loss jointly accounted for more than half of the associations with depression and anxiety.

**Meaning:**

In this study, wildfire exposure and its socioeconomic aftermath were linked to widespread graded psychological harms, underscoring the need to incorporate mental health care, stable housing, and economic recovery into disaster response frameworks.

## Teaser

This cross-sectional study conducted among Maui residents examines associations between wildfire exposure following the 2023 Maui wildfires and symptoms of depression, anxiety, and suicidal ideation.

## Introduction

As climate-related disasters escalate in frequency and intensity, their mental health implications represent an urgent yet underquantified dimension of global health.^1,2^ Extreme events, including wildfires,^3^ hurricanes,^4,5,6,7,8^ and floods,^9,10^ are consistently associated with elevated rates of depression, anxiety, posttraumatic stress disorder, and suicidality, particularly among individuals with direct exposure or preexisting social vulnerabilities.^11,12^ However, most existing evidence relies on cross-sectional designs, convenience samples, or administrative records that may undercapture undiagnosed psychological distress—especially in rural and underserved populations with limited access to care. These methodological limitations restrict robust inference and obscure the true burden of disaster-related mental illness. Despite increasing global attention to this issue, population-based studies using rigorous analytic frameworks to evaluate disaster-related mental health in high-risk, structurally vulnerable US communities remain exceedingly rare.

The 2023 Maui wildfires—one of the deadliest in the US in more than a century—offer a critical case study. The fires devastated the town of Lahaina and surrounding communities, killing more than 100 people and destroying more than 2200 structures. Approximately 10 000 residents were displaced, with many losing their homes, employment, access to essential services, and social support networks.^13^ Recovery unfolded in a geographically isolated and racially and ethnically diverse setting already burdened by longstanding inequities in housing and mental health care, compounding the psychological strain. Although commentaries^14,15^ have underscored likely long-term distress among displaced residents and Native Hawaiian communities, quantitative, population-level evidence has been lacking.

To address these gaps, this study used a cross-sectional, propensity score–weighted design to examine the associations between wildfire exposure, housing displacement, and employment disruption and mental health symptoms among a large, multilingual sample of Maui residents. Using data from the Maui Wildfire Exposure Study (MauiWES)^16^ and a demographically matched statewide comparison cohort from the UHERO Rapid Health Survey (UHERO-RHS),^17^ we evaluated how geographic proximity to burn zones and postdisaster socioeconomic instability relate to depression, anxiety, and suicidal ideation.

This analysis expands on prior work from MauiWES,^16^ which previously described midterm respiratory and psychological outcomes in a single-arm descriptive framework. The current study extends that work by expanding the cohort, introducing a population-based comparison group, and applying propensity score weighting to estimate relative risks, evaluate indirect pathways through displacement and income loss, and assess community-wide spillover effects. Collectively, these advances provide one of the first analytically weighted, population-based assessments of wildfire-related psychological distress in a high-risk US population, with implications for climate adaptation policy, trauma-informed disaster recovery, and equitable mental health response systems worldwide.

## Methods

### Study Design, Participants, and Ethical Considerations

This cross-sectional study assessed associations between wildfire exposure and mental health outcomes after the August 2023 Maui wildfires using 2 complementary sources: MauiWES (n = 1535) and the fifth wave of UHERO-RHS (n = 918). Both studies surveyed adults aged 18 years or older. MauiWES provided exposed participants; UHERO-RHS participants residing outside Maui served as unexposed controls. The final analytic sample included 2453 adults after excluding those with missing mental health or demographic data, as described in the Consolidated Standards of Reporting Trials (CONSORT)–style participant flow diagram (eFigure 1 in Supplement 1). The University of Hawaiʻi institutional review board approved the study, and written informed consent was obtained from all participants. Reporting followed Strengthening the Reporting of Observational Studies in Epidemiology (STROBE) reporting guidelines for observational studies and the AGReMA (A Guideline for Reporting Mediation Analyses) short-form checklist.^18^

### MauiWES Cohort (Exposed Group)

MauiWES is the largest disaster-related biosocial cohort in Hawai‘i, designed to evaluate intermediate and long-term health effects.^16^ Between January 2024 and February 2025 (6-18 months postfire, see eFigure 2 in Supplement 1), participants were enrolled through community-based, multilingual recruitment at shelters, hotel housing sites, and screening events. Materials and interpreter support were provided in English, Spanish, Tagalog, Ilocano, and Pacific Islander languages. Baseline assessments included validated mental health screening, clinical measures, and biospecimen collection (see the eAppendix in Supplement 1).

### UHERO-RHS Cohort (Unexposed Control Group)

UHERO-RHS is a statewide longitudinal biennial survey that monitors health and social disparities across Hawaiʻi.^17^ For this study, participants were restricted to those living outside Maui County. Data were collected in fall 2024 using the same validated mental health instruments as MauiWES, thus ensuring comparability. Mental health measures in UHERO-RHS have remained stable across survey waves, providing a credible reference group.^19^

### Exposure Classification

Residential addresses at the time of the fires were geocoded and linked to burn perimeter shapefiles for the Lahaina and Kula wildfires.^20,21^ Individuals were classified into the following 3 mutually exclusive exposure groups: (1) burn zone, comprising residents whose primary address fell within an official burn perimeter; (2) outside burn zone, comprising Maui residents living elsewhere on the island; and (3) unexposed controls, comprising residents of other Hawaiian Islands from the UHERO-RHS. This classification enabled assessment of dose-response patterns in mental health by degree of exposure.

### Measures

#### Outcomes and Covariates

Three primary mental health outcomes were assessed. Depression was measured with the 10-item Center for Epidemiologic Studies Depression Scale (CES-D; score ≥10 for clinically significant).^22^ Anxiety was measured with the 7-item Generalized Anxiety Disorder scale (GAD-7; score ≥10 for moderate to severe).^23^ Suicidal ideation was assessed using a single self-report item on serious consideration of suicide in the past 30 days, consistent with US Centers for Disease Control and Prevention surveys.^24,25^

Analyses adjusted for demographic and socioeconomic covariates linked to disaster-related mental health.^17^ Matching variables included age, sex, education, race and ethnicity, and Area Deprivation Index (ADI). Employment (employed, unemployed, retired) and housing status (original, temporary, new home) were added as postdisaster covariates. In mediation models, income change and housing displacement were evaluated as mediators. Full metrics and variable definitions are provided in the eAppendix in Supplement 1.

### Statistical Analysis

Associations between wildfire exposure and mental health outcomes were estimated using a multiarm inverse probability of treatment weighting (IPTW) approach based on generalized boosted models.^26^ This propensity score framework balanced covariates across the 3 exposure groups, enabling estimation of average treatment effects (ATE) with reduced confounding.^27^

To improve comparability between exposed and unexposed participants, weighting was based on pre-exposure, time-invariant characteristics (age, sex, race and ethnicity, education), and neighborhood socioeconomic disadvantage, measured using the ADI for each participant’s census block group from 2020 (before the wildfire).^28,29,30^ The ADI is a composite of 17 indicators of income, education, employment, and housing quality that provides a standardized, census-based measure of neighborhood socioeconomic context across US populations. Its inclusion helps reduce structural and regional confounding between Maui and other islands. The variation in ADI score is presented in eFigure 6 in Supplement 1. Participants outside the region of common support were excluded to ensure balance and overlap (caliper = 0.1).

Primary associations between wildfire exposure and mental health outcomes were estimated using weighted logistic regression models with a doubly robust specification, adjusting for both IPTW weights and residual covariate imbalances. This approach improves estimate precision and mitigates bias due to model misspecification.

Total effects of wildfire exposure were decomposed into natural direct and indirect components operating through housing displacement and income-related employment disruption. To explore potential mediating pathways, we conducted survey-weighted structural equation modeling to quantify indirect effects of displacement and employment disruption (proxied by income decrease) on psychological outcomes.^31^ Indirect, direct, and total effects were estimated simultaneously and reported with their 95% confidence intervals.

Causal interpretation of the mediation effects assumes (1) no unmeasured confounding of exposure-mediator, mediator-outcome, or exposure-outcome relations; (2) no mediator-outcome confounders affected by exposure; and (3) correct model specification. Potential violations of these assumptions are discussed in the Limitations section. Mediators and outcomes were measured concurrently 6 to 18 months postwildfire.

Robustness of findings was assessed through multiple sensitivity analyses, including estimating the ATE on the treated using IPTW restricted to exposed subgroups^32^ and conducting pairwise nearest-neighbor matching within a 0.1-caliper region of common support to validate comparability across exposure levels.^33^ All analyses were conducted in R version 4.4.2 (R Foundation for Statistical Computing) using the twang, MatchIt, survey, and lavaan.survey packages. All *P* values were 2-sided, with statistical significance defined as *P* < .05.

## Results

### Baseline Demographic Characteristics and Covariate Balance

The Table summarizes baseline demographic and socioeconomic characteristics across exposure groups (unexposed controls, residents within wildfire burn zones, and residents outside burn zones). The analytic sample included 2453 adults (1535 wildfire exposed and 918 unexposed), among whom mean (SD) age was 50.8 (16.3) years and 1502 participants (61.2%) were women. Before weighting, groups differed meaningfully in age, sex, race and ethnicity, education, and employment status, with unexposed controls being older and more likely to identify as Asian. After applying ATE weighting based on pre-exposure characteristics, these baseline covariates were well balanced across groups (eFigure 3 in Supplement 1). Outcome comparisons presented below are based on the weighted analytic sample.

**Table.** Characteristics of Study Participants Before and After Weighting by Wildfire Exposure Group

Characteristic	No. (%)^a^						
	Overall (N = 2453)	Unweighted sample			Weighted sample		
		Burn zone (n = 658)	Outside burn zone (n = 877)	Unexposed controls (n = 918)	Burn zone (n = 492.69)	Outside burn zone (n = 350.19)	Unexposed controls (n = 508.65)
Outcomes							
Depression							
No problem	1432 (58.38)	303 (46.05)	470 (53.59)	659 (71.79)	229 (46.41)	199 (56.82)	367 (72.17)
Depressive or highly depressive symptom	1021 (41.62)	355 (53.95)	407 (46.41)	259 (28.21)	264 (53.59)	151 (43.18)	142 (27.83)
Anxiety							
Minimal or mild anxiety	1954 (79.66)	455 (69.15)	686 (78.22)	813 (88.56)	343 (69.64)	283 (80.77)	450 (88.45)
Moderate or severe anxiety	499 (20.34)	203 (30.85)	191 (21.78)	105 (11.44)	150 (30.36)	67 (19.23)	59 (11.55)
Suicidal ideation							
No	2380 (97.02)	629 (95.59)	845 (96.35)	906 (98.69)	473 (95.95)	339 (96.76)	504 (99.03)
Yes	73 (2.98)	29 (4.41)	32 (3.65)	12 (1.31)	20 (4.05)	11 (3.24)	5 (0.97)
Covariates							
Age, mean (SD), y	50.80 (16.29)	48.89 (15.64)	46.91 (15.51)	55.88 (16.16)	49.75 (16.63)	49.01 (14.95)	51.32 (16.02)
Gender							
Female	1502 (61.23)	379 (57.60)	556 (63.40)	567 (61.76)	303 (61.44)	225 (64.28)	320 (62.94)
Male	951 (38.77)	279 (42.40)	321 (36.60)	351 (38.24)	190 (38.56)	125 (35.72)	189 (37.06)
Education							
Some schooling, no high school diploma	207 (8.44)	104 (15.81)	94 (10.72)	9 (0.98)	47 (9.58)	30 (8.64)	23 (4.43)
High school diploma	505 (20.59)	179 (27.20)	236 (26.91)	90 (9.80)	112 (22.77)	72 (20.61)	89 (17.49)
Some college level or technical or vocational degree	713 (29.07)	196 (29.79)	269 (30.67)	248 (27.02)	149 (30.33)	104 (29.71)	148 (29.18)
Bachelor’s degree or higher	1028 (41.91)	179 (27.20)	278 (31.70)	571 (62.20)	184 (37.32)	144 (41.04)	249 (48.9)
Race and ethnicity^b^							
Asian							
No	1670 (68.08)	539 (81.91)	724 (82.55)	407 (44.34)	357 (72.5)	252 (72.06)	326 (64.13)
Yes	783 (31.92)	119 (18.09)	153 (17.45)	511 (55.66)	136 (27.5)	98 (27.94)	182 (35.87)
Filipino							
No	1920 (78.27)	508 (77.20)	638 (72.75)	774 (84.31)	378 (76.74)	271 (77.48)	398 (78.27)
Yes	533 (21.73)	150 (22.80)	239 (27.25)	144 (15.69)	115 (23.26)	79 (22.52)	111 (21.73)
Hispanic or Latino							
No	2164 (88.22)	529 (80.40)	753 (85.86)	882 (96.08)	430 (87.14)	309.19 (88.24)	482.65 (94.88)
Yes	289 (11.78)	129 (19.60)	124 (14.14)	36 (3.92)	63 (12.86)	41 (11.76)	26 (5.12)
Native Hawaiian or Pacific Islander							
No	1923 (78.39)	528 (80.24)	653 (74.46)	742 (80.83)	385 (78.21)	277 (79.23)	386 (75.93)
Yes	530 (21.61)	130 (19.76)	224 (25.54)	176 (19.17)	107 (21.79)	73 (20.77)	122 (24.07)
White							
No	1439 (58.66)	392 (59.57)	510 (58.15)	537 (58.50)	283 (57.45)	194 (55.35)	279 (54.78)
Yes	1014 (41.34)	266 (40.43)	367 (41.85)	381 (41.50)	210 (42.55)	156 (44.65)	230 (45.22)
Other race^c^							
No	2292 (93.44)	601 (91.34)	805 (91.79)	886 (96.51)	449 (91.1)	322 (91.93)	492 (96.78)
Yes	161 (6.56)	57 (8.66)	72 (8.21)	32 (3.49)	44 (8.9)	28 (8.07)	16 (3.22)
House relocation							
No	1652 (67.35)	89 (13.53)	647 (73.77)	916 (99.78)	81 (16.53)	268 (76.48)	508 (99.86)
Yes	801 (32.65)	569 (86.47)	230 (26.23)	2 (0.22)	411 (83.47)	82 (23.52)	1 (0.14)
Employment status							
Employed	1488 (60.66)	354 (53.80)	597 (68.07)	537 (58.50)	260 (52.69)	247 (70.47)	338 (66.38)
Retired	454 (18.51)	77 (11.70)	99 (11.29)	278 (30.28)	76 (15.48)	42 (12.01)	112 (21.93)
Unemployed	511 (20.83)	227 (34.50)	181 (20.64)	103 (11.22)	157 (31.83)	61 (17.52)	59 (11.7)
Area Deprivation Index, mean (SD)	5.20 (2.67)	5.57 (2.43)	4.83 (2.35)	5.29 (3.05)	5.46 (2.35)	5.21 (2.51)	5.15 (2.76)

^a^ This Table presents continuous variables and categorical variables of key sociodemographic characteristics and mental health outcomes across treatment groups in both the unweighted and average treatment effect (ATE)–weighted analytic samples. Treatment groups include: (1) unexposed controls (non-Maui residents), (2) residents outside the wildfire burn zone, and (3) residents within the burn zone. The ATE-weighted sample included a total effective sample size of 1351.53 individuals: 508.65 in the unexposed group, 350.19 outside the burn zone, and 492.69 within the burn zone. Propensity score weighting was performed using generalized boosted models to achieve covariate balance across age, gender, education, race and ethnicity (Asian, Filipino, Hispanic or Latino, Native Hawaiian or Pacific Islander, or White), and Area Deprivation Index. After weighting, the treatment and unexposed controls were well balanced across baseline covariates.

^b^ Participants were asked to self-identify their racial or ethnic group through the question “Which 1 or more of the following would you say is your race?” For more details, see the eAppendix in Supplement 1.

^c^ Other races include self-reported Black or African American, American Indian or Alaska Native, and other race or ethnicity.

### Mental Health Outcomes by Wildfire Exposure

**Figure 1.**
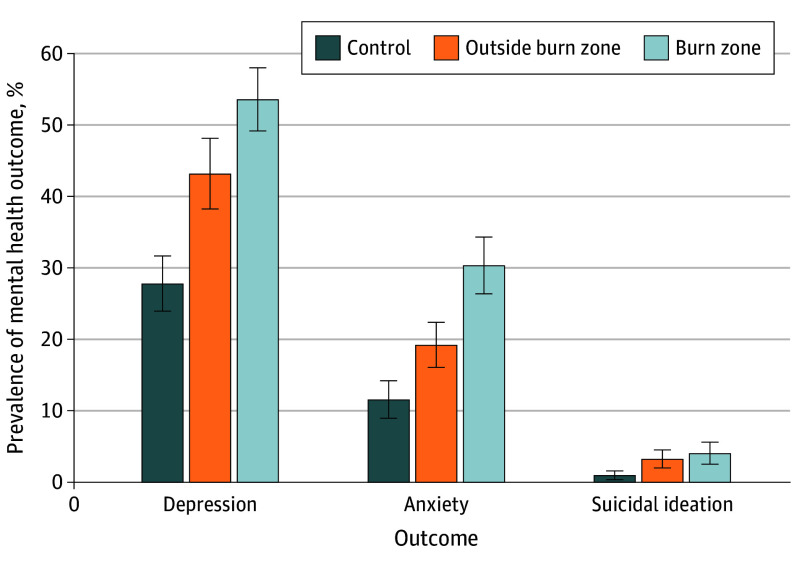
Bar Graph Showing Weighted Prevalence of Mental Health Outcomes by Wildfire Exposure Group Bar graphs show the weighted prevalence (%) of depressive symptoms, anxiety symptoms, and suicidal ideation across 3 exposure groups: unexposed controls (dark blue), Maui residents outside the wildfire burn zones (orange), and residents within burn zones (light blue). Prevalence of all outcomes increased with proximity to the burn zones, consistent with a dose-response pattern. Error bars indicate 95% confidence intervals.

Before weighting, the prevalence of adverse mental health outcomes varied substantially by exposure group. Clinically significant depression symptoms were reported by 259 of 918 unexposed controls (28.21%), 355 of 658 burn zone residents (53.95%), and 407 of 877 residents outside burn zones (46.41%). Clinically significant anxiety symptoms followed a similar gradient: 105 of 918 among unexposed controls (11.44%), 203 of 658 residents in the burn zone (30.85%), and 191 of 877 residents outside the burn zone (21.78%). Suicidal ideation was reported by 12 of 918 unexposed controls (1.31%), 29 of 658 burn zone residents (4.41%), and 32 of 877 among those outside burn zones (3.65%). After applying ATE weights, group differences persisted. Weighted prevalence of depression was 142 of 509 among unexposed controls (27.83%; 95% CI, 23.99%-31.66%), 264 of 493 among burn zone residents (53.59%; 95% CI, 49.16%-58.03%), and 151 of 350 among residents outside burn zones (43.18%; 95% CI, 38.24%-48.12%). Weighted anxiety prevalences were 59 of 509 (11.55%; 95% CI, 8.94%-14.17%), 150 of 493 (30.36%; 95% CI, 26.41%-34.30%), and 67 of 350 (19.23%; 95% CI, 16.06%-22.39%), respectively. Weighted suicidal ideation remained most frequent among burn zone residents (20 of 493; 4.05%; 95% CI, 2.51%-5.59%) and residents outside burn zones (11 of 350; 3.24%; 95% CI, 1.98%-4.49%) relative to unexposed controls (5 of 509; 0.97%; 95% CI, 0.35%-1.59%) (Figure 1).

As illustrated in eFigures 4 and 5 in Supplement 1, both depression and anxiety scores demonstrated a dose-response gradient, with symptom severity increasing in proportion to wildfire exposure.

### Weighted Associations Between Wildfire Exposure and Mental Health Outcomes

**Figure 2.**
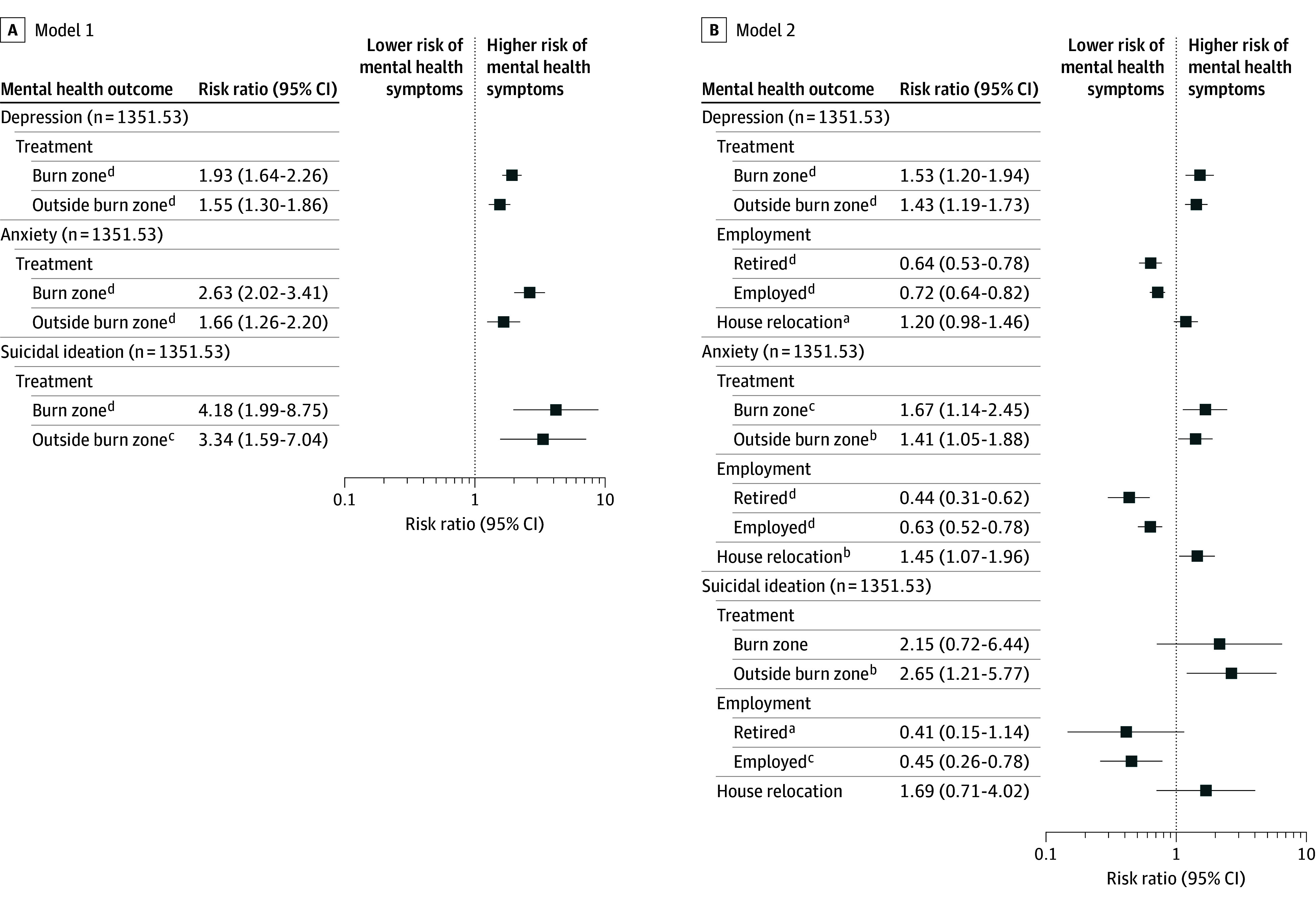
Forest Plots Showing Adjusted Risk Ratios for Mental Health Outcomes by Wildfire Exposure and Social Determinants Log-scale forest plots show adjusted risk ratios with 95% confidence intervals for depression, anxiety, and suicidal ideation across 2 models. Model 1 (A) includes wildfire exposure. Model 2 (B) further adjusts for employment status and housing relocation. Results are based on average treatment effect weighting using a weighted analytic sample (N = 1351.53), comprising 508.65 weighted unexposed controls, 492.69 burn zone residents, and 350.19 residents outside the burn zone. The vertical reference line at risk ratio = 1 denotes no association. Risk ratio >1 indicates higher risks of mental health symptoms; risk ratio <1 indicates lower risks. Full regression results are reported in eTable 1 in Supplement 1. ^a^*P* < .10. ^b^*P* < .05. ^c^*P* < .01. ^d^*P* < .001.

Wildfire exposure was significantly associated with increased risk of adverse mental health outcomes (Figure 2). In fully adjusted, propensity-weighted models, residents within wildfire burn zones had higher risk of depressive symptoms (risk ratio [RR], 1.53; 95% CI, 1.20-1.94) and moderate to severe anxiety (RR, 1.67; 95% CI, 1.14-2.45) compared with unexposed controls. Although suicidal ideation was more frequent among burn zone residents (RR, 2.15; 95% CI, 0.72-6.44), this association was not statistically significant.

Maui residents living outside burn zones also exhibited significantly higher risk of depression (RR, 1.43; 95% CI, 1.19-1.73), anxiety (RR, 1.41; 95% CI, 1.05-1.88), and suicidal ideation (RR, 2.65; 95% CI, 1.21-5.77) relative to unexposed individuals, indicating community-wide psychological effects extending beyond directly affected areas.

Structural and socioeconomic disruptions were associated with psychological distress. Participants residing in temporary housing had a significantly higher risk of anxiety (RR, 1.45; 95% CI, 1.07-1.96) and a higher, approaching statistical significance risk of depression (RR, 1.20; 95% CI, 0.98-1.46) compared with those in stable housing.

In contrast, employment status emerged as a strong protective factor against mental health outcomes: being employed was associated with substantially lower risk of depression (RR, 0.72; 95% CI, 0.64-0.82), anxiety (RR, 0.63; 95% CI, 0.52-0.78), and suicidal ideation (RR, 0.45; 95% CI, 0.26-0.78) (Figure 2; eTable 1 in Supplement 1).

### Indirect Pathways Linking Wildfire Exposure to Mental Health Symptoms

To explore pathways through which wildfire exposure was associated with psychological outcomes, we conducted mediation analyses using survey-weighted structural equation modeling. Two mediators were examined—housing displacement and employment disruption (proxied by income decrease)—each hypothesized to transmit part of the association between exposure and mental health symptoms.

**Figure 3.**
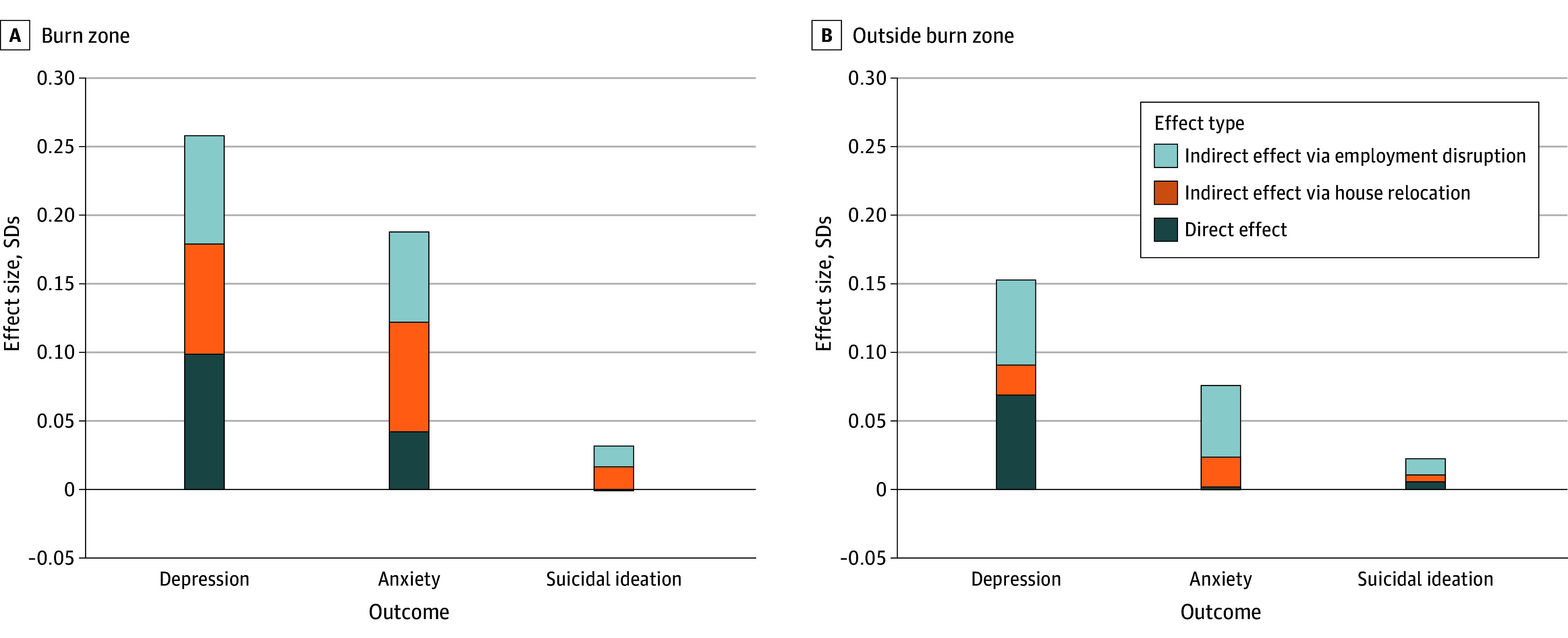
Bar Graph Showing Direct and Indirect Effects of Wildfire Exposure on Mental Health Outcomes Standardized coefficients from a survey-weighted structural equation model show total, direct, and indirect effects of wildfire exposure on mental health outcomes. Indirect effects are mediated through 2 pathways: housing instability and employment disruption (proxied by income decrease). Residents in the burn zone (A) experienced significant direct and indirect increases in depressive symptom burden, with housing displacement and employment disruption equally emerging as the mediators, compared to residents outside the burn zone (B). Full model results are reported in eTable 2 in Supplement 1.

Among residents within the wildfire burn zone, exposure was associated with a significantly higher latent propensity for depressive symptoms relative to unexposed controls (total effect: β = 0.258; 95% CI, 0.198-0.317). Decomposition of the total effect indicated contributions from a direct pathway (β = 0.099; 95% CI, −0.003 to 0.201) and indirect pathways through housing displacement (β = 0.080; 95% CI, 0.005-0.156) and employment disruption (β = 0.079; 95% CI, 0.036-0.121), which jointly mediated the remainder. Combined, these mediators explained approximately 61.62% of the total wildfire effect on depressive symptoms and 77.66% on anxiety. Outside the burn zone, the corresponding proportions mediated were 54.54% for depression and 96.10% for anxiety. For suicidal ideation in the burn zone, the estimated direct effect was negative and the proportion mediated exceeded 100%, consistent with suppression or inconsistent mediation. Full mediation results for all mental health outcomes and exposure groups are presented in Figure 3 and eTable 2 in Supplement 1.

### Sensitivity and Supplementary Analyses

To assess the robustness of results, multiple sensitivity and supplementary analyses were performed using alternative weighting, matching, and population adjustments (eTables 3-9 in Supplement 1).

First, we re-estimated associations using ATE on the treated weights and pairwise nearest-neighbor matching restricted to participants within common support regions (eFigure 8, eTables 3 and 4 in Supplement 1). Both approaches produced results consistent with the primary weighted models, showing that direct and indirect wildfire exposure remained associated with higher prevalence of depression, anxiety, and suicidal ideation (eFigure 7, eTable 5 in Supplement 1). Pairwise matching reinforced that residents within burn zones experienced the highest mental health burden (eTable 6 in Supplement 1).

Second, to test generalizability to the broader state population, poststratification weights were applied based on the Hawaiʻi state population from the American Community Survey. ATE estimates remained consistent with primary results (eTable 7 in Supplement 1).

Third, to examine temporal stability, analyses were replicated using the third wave of UHERO-RHS, collected before the fires, as an unexposed reference group. Associations between wildfire exposure and both depression and suicidal ideation remained unchanged (eTable 8 in Supplement 1).

Finally, sensitivity analyses using E-values^34^ indicated that the associations of wildfire exposure with depression and anxiety were moderately robust to unmeasured confounding. In contrast, the association with suicidal ideation was not statistically significant (eTable 9 in Supplement 1).

Across all sensitivity and supplementary analyses, results were consistent in magnitude and direction, supporting the robustness of associations for depression and anxiety, while effects on suicidal ideation were less stable.

## Discussion

The 2023 Maui wildfires—among the deadliest in modern US history—underscore the severe mental health toll of climate-related disasters. In this racially and ethnically diverse, geographically isolated cohort, wildfire exposure—particularly when accompanied by housing or income disruption—was linked to higher prevalence of depression, anxiety, and suicidal ideation and was not limited to only those directly impacted.

These findings extend prior reports of cardiopulmonary and psychological effects among directly exposed residents,^16^ rising suicide deaths,^35^ and crisis line calls.^36^ Differences between our findings and prior reports of increased suicide mortality likely reflect differences in outcome definition, timing, and level of analysis. Prior work examined county-level suicide deaths during the acute wildfire period, whereas our study assessed individual-level, 30-day suicidal ideation 6 to 18 months postfire, a relatively infrequent outcome in population surveys, which limits precision due to small numbers of positive responses. Together, they depict a consistent pattern of heightened distress across clinical and community settings. Our analysis advances this evidence by quantifying population-level excess risk, identifying modifiable mediators (housing displacement and income loss), and showing that psychological distress extended beyond the burn zone.

Distress followed a graded, dose-response pattern, with the greatest burden among burn zone residents and attenuated yet significant effects among neighbors. Associations persisted after IPTW adjustment, suggesting they are unlikely to be due to confounding alone. Mediation analyses indicate that housing and employment disruption jointly explained much of the observed increase in depression and anxiety, highlighting that social and economic instability—rather than trauma exposure alone—drives population-level harm. Strengthening housing stability, employment continuity, and community cohesion may mitigate long-term impacts, consistent with a syndemic framework in which environmental shocks amplify social vulnerability.

Unlike administrative mortality or service utilization data,^35,36,37^ our community-based screening captures clinically significant symptoms (including among displaced, uninsured, and multilingual residents) that are often undiagnosed and therefore not observable in administrative sources. The findings show that trauma-related symptoms extend beyond physical destruction, driven by community loss, housing instability, and economic disruption.

While disaster research often centers on posttraumatic stress disorder, our results suggest that depression, anxiety, and suicidality linked to socioeconomic disruption are more widespread, immediate, and modifiable and should be treated as core components of disaster recovery. Beyond social pathways, biological mechanisms may also contribute: wildfire smoke exposure is associated with systemic inflammation, oxidative stress, and neurobiological changes tied to depression and suicidality.^38,39,40^ Future work integrating biomarker analysis from the MauiWES biorepository will clarify how environmental exposures interact with psychosocial stress to shape individual mental health trajectories.

The findings reinforce global calls to integrate mental health into climate resilience planning. Frameworks like the Sendai Framework for Disaster Risk Reduction,^41^ the *Lancet* Countdown on Health and Climate Change,^42^ and World Health Organization climate adaptation guidance^43^ all emphasize psychological well-being as essential to preparedness and recovery. This study offers rare empirical evidence for that agenda, showing that mental health is core disaster infrastructure, not secondary to physical reconstruction.

In Hawaiʻi, where housing costs are among the nation’s highest and social supports are limited, these results underscore the need to pair long-term housing solutions with trauma-informed mental health care. Evidence-based, trauma-specific interventions—such as trauma-focused cognitive behavioral therapy and culturally grounded, community-based counseling—can be embedded within disaster response systems to address both acute and chronic distress. Transitional shelters alone are insufficient; recovery must treat the psychological toll of displacement as a central pillar of resilience.

Globally, as climate disasters intensify, few countries monitor or mitigate their mental health impacts. These findings demonstrate the feasibility and necessity of embedding mental health surveillance and trauma-informed interventions within disaster preparedness frameworks to identify vulnerable populations early and guide equitable recovery.

### Strengths and Limitations

This study offers several methodological strengths. To our knowledge, it is among the first to apply multiarm propensity score weighting with a contemporaneous control group to estimate the mental health effects of wildfire exposure. Participants were matched not only on individual demographic characteristics, but also on neighborhood-level deprivation indices, reducing confounding and strengthening inference.

However, several limitations warrant consideration. Residual confounding from unmeasured factors—such as prior trauma, informal social support, or undiagnosed psychiatric conditions—cannot be ruled out. Regional differences in health care access or social cohesion may also contribute to bias despite adjustment using the ADI, a validated measure of neighborhood disadvantage. Because the ADI is derived from national socioeconomic indicators, it may underestimate contextual deprivation in Hawai‘i communities where high living costs, geographic isolation, and limited clinician availability are not fully captured. Notably, estimates were similar in magnitude and direction in sensitivity analyses conducted without adjustment for ADI. Selection bias is possible due to community-based recruitment, although it would likely attenuate rather than exaggerate group differences. Some variables, including housing and employment, functioned as both mediators and potential confounders, complicating interpretation. Mental health outcomes were self-reported, introducing possible recall or reporting bias, and data collected 6 to 18 months postfire cannot capture longer-term trajectories, although this period aligns with the peak prevalence of postdisaster distress observed in prior research.^1,44,45,46^ Suicidal ideation was assessed using a single-item measure, which, while common in population-based studies, does not capture the multidimensional nature of suicidality; more comprehensive instruments should be considered in future work to strengthen measurement validity. Notably, associations involving suicidal ideation were less precise, likely reflecting limited power due to the low event frequency rather than the absence of an effect.

Interpretation of mediation findings depends on standard causal inference assumptions (no unmeasured confounding and correct model specification); concurrent measurement of mediators and outcomes limits temporal ordering. Exposure classification based on burn zone residence may not fully reflect individual pollutant exposure or fire intensity, but Maui’s small geographic scale likely minimizes misclassification.

Despite these limitations, the community-based design, representative sampling, and validated screening instruments strengthen internal validity and enhance generalizability to underserved and rural populations often missing from administrative datasets.

## Conclusions

In conclusion, the 2023 Maui wildfires illustrate that climate-related disasters are associated with substantial and far-reaching mental health impacts. In this cross-sectional study, both direct and indirect exposure were associated with higher prevalence of depression, anxiety, and suicidal ideation, magnified by housing displacement and economic disruption. These findings show that psychological distress extends beyond physical burn zones, reflecting a community-wide toll.

Housing stability and employment continuity emerged as key modifiable determinants of postdisaster well-being. Effective recovery must therefore move beyond physical reconstruction to integrate mental health care, stable housing, and economic security as core components of disaster response. As climate extremes intensify, proactive surveillance, coordinated services, and culturally grounded care will be essential to rebuilding not only infrastructure, but also the psychological and social fabric of affected communities.

## Notes

Supplement 1. eAppendix. Study Description and Eligibility Criteria, Variable Description, and Sampling and Recruitment eFigure 1. Data Cleaning and Flow Diagram eFigure 2. Monthly Recruitment Distribution for the Maui Wildfire Exposure Study, January 2024-February 2025 eFigure 3. Covariate Balance Before and After Propensity Score Weighting eFigure 4. Distribution of Mental Health Scores by Wildfire Exposure Group eFigure 5. Geographic Variation in Mental Health Scores by ZIP Code eFigure 6. Geographic Variation in Area Deprivation Index by Census Block Group in 2020 eTable 1. Association Between Wildfire Exposure and Mental Health Outcomes Using Propensity Score–Weighted Models eTable 2. Mediation of Wildfire Exposure Effects on Mental Health Outcomes via Housing Relocation and Employment Disruption eFigure 7. Sensitivity Analysis: Effects of Wildfire Exposure on Mental Health Using ATT Weighting eFigure 8. Distribution of Wildfire Exposure Propensity Scores by Treatment Group eTable 3. Covariate Balance by Treatment Group in Unweighted and ATT-Weighted Samples eTable 4. Covariate Balance Across Treatment Groups Using Pairwise Propensity Score Matching eTable 5. Association Between Wildfire Exposure and Mental Health Outcomes Using ATT Weighting eTable 6. Effects of Wildfire Exposure on Mental Health Outcomes Using Pairwise Propensity Score Matching eTable 7. Association Between Wildfire Exposure and Mental Health Outcomes in State Population-Weighted Sample Using Multi-Arm Propensity Score Matching eTable 8. Association Between Wildfire Exposure and Mental Health Outcomes Using Pre-Wildfire Controls From UHERO-RHS Wave 3 eTable 9. Robustness Check With E-values for the Association Between Wildfires Exposure and Mental Health Outcomes

Supplement 2. Data Sharing Statement
