# Supplementary material for: Housing Displacement, Employment Disruption, and Mental Health After the 2023 Maui Wildfires
Source: JAMA Psychiatry. 2026 Mar 11;83(6):601–10. doi: 10.1001/jamapsychiatry.2026.0044 (PMC12980362; doi:10.1001/jamapsychiatry.2026.0044)
Supplement: Supplement 1. — eAppendix. Study Description and Eligibility Criteria, Variable Description, and Sampling and Recruitment eFigure 1. Data Cleaning and Flow Diagram eFigure 2. Monthly Recruitment Distribution for the Maui Wildfire Exposure Study, January 2024-February 2025 eFigure 3. Covariate Balance Before and After Propensity Score Weighting eFigure 4. Distribution of Mental Health Scores by Wildfire Exposure Group eFigure 5. Geographic Variation in Mental Health Scores by ZIP Code eFigure 6. Geographic Variation in Area Deprivation Index by Census Block Group in 2020 eTable 1. Association Between Wildfire Exposure and Mental Health Outcomes Using Propensity Score–Weighted Models eTable 2. Mediation of Wildfire Exposure Effects on Mental Health Outcomes via Housing Relocation and Employment Disruption eFigure 7. Sensitivity Analysis: Effects of Wildfire Exposure on Mental Health Using ATT Weighting eFigure 8. Distribution of Wildfire Exposure Propensity Scores by Treatment Group eTable 3. Covariate Balance by Treatment Group in Unweighted and ATT-Weighted Samples eTable 4. Covariate Balance Across Treatment Groups Using Pairwise Propensity Score Matching eTable 5. Association Between Wildfire Exposure and Mental Health Outcomes Using ATT Weighting eTable 6. Effects of Wildfire Exposure on Mental Health Outcomes Using Pairwise Propensity Score Matching eTable 7. Association Between Wildfire Exposure and Mental Health Outcomes in State Population-Weighted Sample Using Multi-Arm Propensity Score Matching eTable 8. Association Between Wildfire Exposure and Mental Health Outcomes Using Pre-Wildfire Controls From UHERO-RHS Wave 3 eTable 9. Robustness Check With E-values for the Association Between Wildfires Exposure and Mental Health Outcomes [file jamapsychiatry-e260044-s001.pdf]

Supplemental Online Content

Juarez R, Le B, Knightsbridge C, Lowery M, Maunakea AK. Housing displacement, employment disruption, and mental health after the 2023 Maui wildfires. *JAMA Psychiatry*. Published online March 11, 2026. doi:10.1001/jamapsychiatry.2026.0044

eAppendix. Study Description and Eligibility Criteria, Variable Description, and Sampling and Recruitment
eFigure 1. Data Cleaning and Flow Diagram
eFigure 2. Monthly Recruitment Distribution for the Maui Wildfire Exposure Study, January 2024-February 2025
eFigure 3. Covariate Balance Before and After Propensity Score Weighting
eFigure 4. Distribution of Mental Health Scores by Wildfire Exposure Group
eFigure 5. Geographic Variation in Mental Health Scores by ZIP Code
eFigure 6. Geographic Variation in Area Deprivation Index by Census Block Group in 2020
eTable 1. Association Between Wildfire Exposure and Mental Health Outcomes Using Propensity Score–Weighted Models
eTable 2. Mediation of Wildfire Exposure Effects on Mental Health Outcomes via Housing Relocation and Employment Disruption
eFigure 7. Sensitivity Analysis: Effects of Wildfire Exposure on Mental Health Using ATT Weighting
eFigure 8. Distribution of Wildfire Exposure Propensity Scores by Treatment Group
eTable 3. Covariate Balance by Treatment Group in Unweighted and ATT-Weighted Samples
eTable 4. Covariate Balance Across Treatment Groups Using Pairwise Propensity Score Matching
eTable 5. Association Between Wildfire Exposure and Mental Health Outcomes Using ATT Weighting
eTable 6. Effects of Wildfire Exposure on Mental Health Outcomes Using Pairwise Propensity Score Matching
eTable 7. Association Between Wildfire Exposure and Mental Health Outcomes in State Population-Weighted Sample Using Multi-Arm Propensity Score Matching
eTable 8. Association Between Wildfire Exposure and Mental Health Outcomes Using Pre-Wildfire Controls From UHERO-RHS Wave 3
eTable 9. Robustness Check With E-Values for the Association Between Wildfires Exposure and Mental Health Outcomes

This supplemental material has been provided by the authors to give readers additional information about their work.

© 2026 Juarez R et al. *JAMA Psychiatry*.

eAppendix. Study Description and Eligibility Criteria, Variable Description, and Sampling and Recruitment

Description of the Study, Variables, and Data Cleaning

Eligibility Criteria Maui Wildfire Exposure Study

Inclusion Criteria

Participants must meet all the following criteria to be eligible for enrollment in the Maui Wildfire Exposure Study (MauiWES):

1. **Age:** Must be an adult at the time of enrollment.
2. **Residency or Employment:** Must have lived or worked within 20 miles of Lahaina, Kula, or any other area directly affected by the August 2023 wildfires.
3. **Language Proficiency:** Must be able to speak and read at least one of the following languages: English, Tagalog, Ilocano, Spanish, Samoan, Marshallese, Micronesian, Tongan, or Chamorro.
4. **Residency Duration & Commitment:** Must have resided in Maui for at least one year before the wildfires and be willing to participate in annual follow-ups for the next five years.
5. **In-Person Participation:** Must be able to attend a community-based event for health assessments, including biometric measurements, biospecimen collection, and a medical consultation.

Exclusion Criteria

Participants will be excluded if they meet any of the following conditions:

1. **Inability or unwillingness to participate**:
    - Unable or unwilling to complete an online questionnaire (or a paper questionnaire upon request).
    - Unable or unwilling to provide biospecimens.
2. **Medical Conditions**:
    - Diagnosed with a medical condition that would prevent full participation (e.g., uncontrolled major psychiatric disorders).
3. **Institutionalization**:
    - Currently residing in an institutional setting, such as a rehabilitation hospital or correctional facility.
4. **Relocation Plans**:
    - Planning to move away from Hawaii within the next 60 months (5 years).

© 2026 Juarez R et al. *JAMA Psychiatry*.

Eligibility Criteria for the UHERO Rapid Survey

Inclusion Criteria

Participants must meet all the following criteria to be eligible for enrollment in the UHERO Rapid Health Survey (UHERO-RHS):

1. **Age:** Must be 18 years of age or older at the time of survey participation.
2. **Residency:** Must currently reside in the State of Hawaiʻi, including any of the counties of Hawaiʻi, Maui, Kauaʻi, or the City and County of Honolulu.
3. **Language Proficiency:** Must be able to read and understand English or another supported language version of the survey or facilitator: English, Tagalog, Ilocano, Spanish, Samoan, Marshallese, Micronesian, Tongan, or Chamorro
4. **Access and Consent:** Must have access to an internet-connected device (e.g., smartphone, tablet, or computer) and be able to provide informed consent electronically.
5. **Unique Participation:** Must agree to participate only once per survey wave to ensure data integrity.

Exclusion Criteria

Participants will be excluded if they meet any of the following conditions:

1. **Non-Residency:** Do not currently reside in Hawaiʻi.
2. **Inability or Unwillingness to Participate:**
   - Unable or unwilling to complete the online questionnaire independently or with minimal assistance.
   - Decline to provide informed consent.
3. **Duplicate or Invalid Entries:**
   - Multiple submissions detected from the same individual or household within a single survey wave.
4. **Institutionalization:**
   - Currently residing in a long-term institutional setting (e.g., correctional facility, inpatient psychiatric or rehabilitation hospital) that prevents independent survey participation.

Variables and Data Cleaning

Outcome variables: We focused on three mental health metrics that included *depression*, *anxiety*, and *suicidal ideation* as follows:

Depression was assessed using the Center for Epidemiological Studies Depression (CES-D) 10-item scale, a self-report tool that measures the frequency of events and ideas related to depression experienced by an individual over the past week. Each item is scored between 0 - "Rarely or none of the time (less than 1 day)," 1- "Some or a little of the time (1-2 days)," 2-

© 2026 Juarez R et al. *JAMA Psychiatry*.

“Occasionally or a moderate amount of time (3-4 days),” and 3- “All of the time (5-7 days).”[1] The questionnaire is as follows:

Below is a list of some of the ways you may have felt or behaved. Please indicate how often you have felt this way during the past week by checking the appropriate box for each row. (Select one option per row)

	Rarely or none of the time (less than 1 day)	Some or a little of the time (1-2 days)	Occasionally or a moderate amount of time (3-4 days)	All of the time (5-7 days)
I was bothered by things that usually don't bother me	○	○	○	○
I had trouble keeping my mind on what I was doing	○	○	○	○
I felt depressed	○	○	○	○
I felt that everything I did was an effort	○	○	○	○
I felt hopeful about the future	○	○	○	○
I felt fearful	○	○	○	○
My sleep was restless	○	○	○	○
I was happy	○	○	○	○
I felt lonely	○	○	○	○
I could not "get going"	○	○	○	○

Anxiety was measured with the Generalized Anxiety Disorder 7-item (GAD-7), which is a commonly used tool to screen anxiety. Each item is scored between 0 - “Not at all,” 1- “Several days,” 2- “More than half the days,” and 3- “Nearly every day.” A total score is between 0 and 21. To simplify, we dichotomized the variable as 1 (“moderate anxiety”/ “severe anxiety” scored 10 or above) and 0 (“minimal anxiety”/ “mild anxiety”). The questionnaire is as follows:

[1] Items 5 and 8 are coded reversely.

© 2026 Juarez R et al. *JAMA Psychiatry*.

Over the last 2 weeks, how often have you been bothered by the following problems?

	Not at all	Several days	Over half the days	Nearly every day
Feeling nervous, anxious, or on edge	○	○	○	○
Not being able to stop or control worrying	○	○	○	○
Worrying too much about different things	○	○	○	○
Trouble relaxing	○	○	○	○
Being so restless that it's hard to sit still	○	○	○	○
Becoming easily annoyed or irritable	○	○	○	○
Feeling afraid as if something awful might happen	○	○	○	○

Suicidal ideation was indicated by a binary variable that equals 1 if the participant reported seriously considering attempting suicide in the past month.

Independent variables

Control variables

We collected **age** as a continuous variable by asking the question, *"What is your year of birth?"*

Participants could report their ***sex*** as *male*, *female*, *other*, or *decline to answer the* question *"What sex was originally listed on your birth certificate?"* The variable is dichotomized as 1 (male) and 0 (female). In our sample, 99.6% of individuals responded male or female.

We collected the ***level of education*** completed by asking the question, *"What is your highest level of education?"* The levels were coded from 1 - "Some schooling, no high school diploma," 2- "High school diploma," 3- "Some college level/Technical/Vocational degree," and 4- "Bachelor's degree or higher."

We asked participants to self-identify their ***race/ethnic group*** through the question "*Which one or more of the following would you say is your race?*" To simplify the analysis, we grouped respondents' choices into one of four major race/ethnic group: NHPI (including Native Hawaiian, Guamanian or Chamorro, Hawaiian, Marshallese, Micronesian, Samoan, and Other Pacific

© 2026 Juarez R et al. *JAMA Psychiatry*.

Islander); Non-Filipino Asian (including Chinese, Japanese, Korean); Filipino; White (including White, Portuguese, Caucasian, and Native American).
To gather information on ***employment status***, we asked participants the question *"What is your current employment status?"* The category variable defined employed ("Full-time"/"Part-time"), unemployed ("Unemployed, not looking for work"/"Seeking opportunities currently"), and retired ("Retired").

House relocation was measured by the question *"Are you currently living in your original home, temporary housing, or a new permanent location?"* The variable is dichotomized as 1 ("Temporary housing"/ "A new permanent location") and 0 ("Original home").

State-ranked Area Deprivation Index (ADI) was collected at the census block-group level to measure the neighborhood disadvantages across population. The values are ranged between 0 and 10. A higher ADI indicates the more disadvantaged neighborhood. Higher ADI is linked to a number of health disparities, including higher levels of adverse social exposome.[2]

Income decrease was measured by the question *"Did your household income change since the wildfires?"* The variable is dichotomized as 1 ("Decreased xx-yy%"/ "No household income since the wildfires")[3] and 0 ("Household income increased since the wildfires"/ "No change").

Wildfire exposure degree was measured by the question *"How frequently have you been exposed to wildfire ash, debris, or smoke?"* The variable is categorized as 0 - "Never"; 1- "Rarely"; 2- "Weekly"; 3- "Daily." The UHERO cohort was coded as having never been exposed to wildfire ash, debris, or smoke.

Sampling and Recruitment

Participants in MauiWES and the UHERO-RHS were recruited through complementary strategies to ensure representativeness and comparability across exposed and unexposed populations.

For MauiWES, 1,837 adults were enrolled between January 2024 and February 2025, corresponding to approximately 6–18 months after the August 2023 wildfires. Recruitment

[2] https://www.neighborhoodatlas.medicine.wisc.edu/
[3] xx-yy% is either less than 10%, 11-20%, 21-30%, ……., 81-89%, 90-99%.

© 2026 Juarez R et al. *JAMA Psychiatry*.

occurred through community-based screening events, hotel shelters, and temporary housing sites, with multilingual outreach in English, Spanish, Tagalog, Ilocano, Samoan, Marshallese, Tongan. After excluding 35 individuals residing outside Maui and 267 with missing covariates or incomplete questionnaires, 1,535 participants were retained in the analytic dataset, representing an attrition rate of 16.4% from the initial registry.

For the UHERO-RHS, 1,114 adults statewide completed Wave 5 (October–December 2024). Of the 1,550 participants recontacted from prior Waves 3 or 4, this represents a completion rate of 71.9%, and after excluding 71 respondents from Maui and those with missing data, an analytic sample of 918 unexposed controls was obtained, corresponding to a complete-case response rate of 82.4% for that wave.

Recruitment for MauiWES relied on voluntary participation from previously identified households and publicly advertised community events held near survivors' housing sites (e.g., hotels, shelters, and community centers), supported by a diverse network of community partners assisting displaced residents. For UHERO-RHS, demographic post-stratification aligned the analytic sample to the 2020 U.S. Census distribution for Hawai'i by age, race, and gender.

A flow diagram of data cleaning, exclusions, and analytic sample derivation is presented in Figure A1 (eAppendix), summarizing the progression from 2,951 initial records to 2,453 complete analytic cases, corresponding to an overall inclusion rate of 83.1%.

© 2026 Juarez R et al. *JAMA Psychiatry*.

eFigure 1. Data Cleaning and Flow Diagram

Our data cleaning protocol is illustrated in the flow diagram. Of 1,114 participants completing the fifth wave UHERO-RHS, 1,043 individuals were included in the sample after removing individuals with zip codes in Maui and outside the state of Hawaii. Of 1,837 participants completing the MauiWES study, 1,802 individuals were included in the sample after removing individuals with zip codes outside of Maui. A total of 2,845 participants were re-cleaned by removing records with missing information on mental health outcomes and control covariates. Removal of missing information was essential for matching. Data from 2,453 individuals was analyzed.

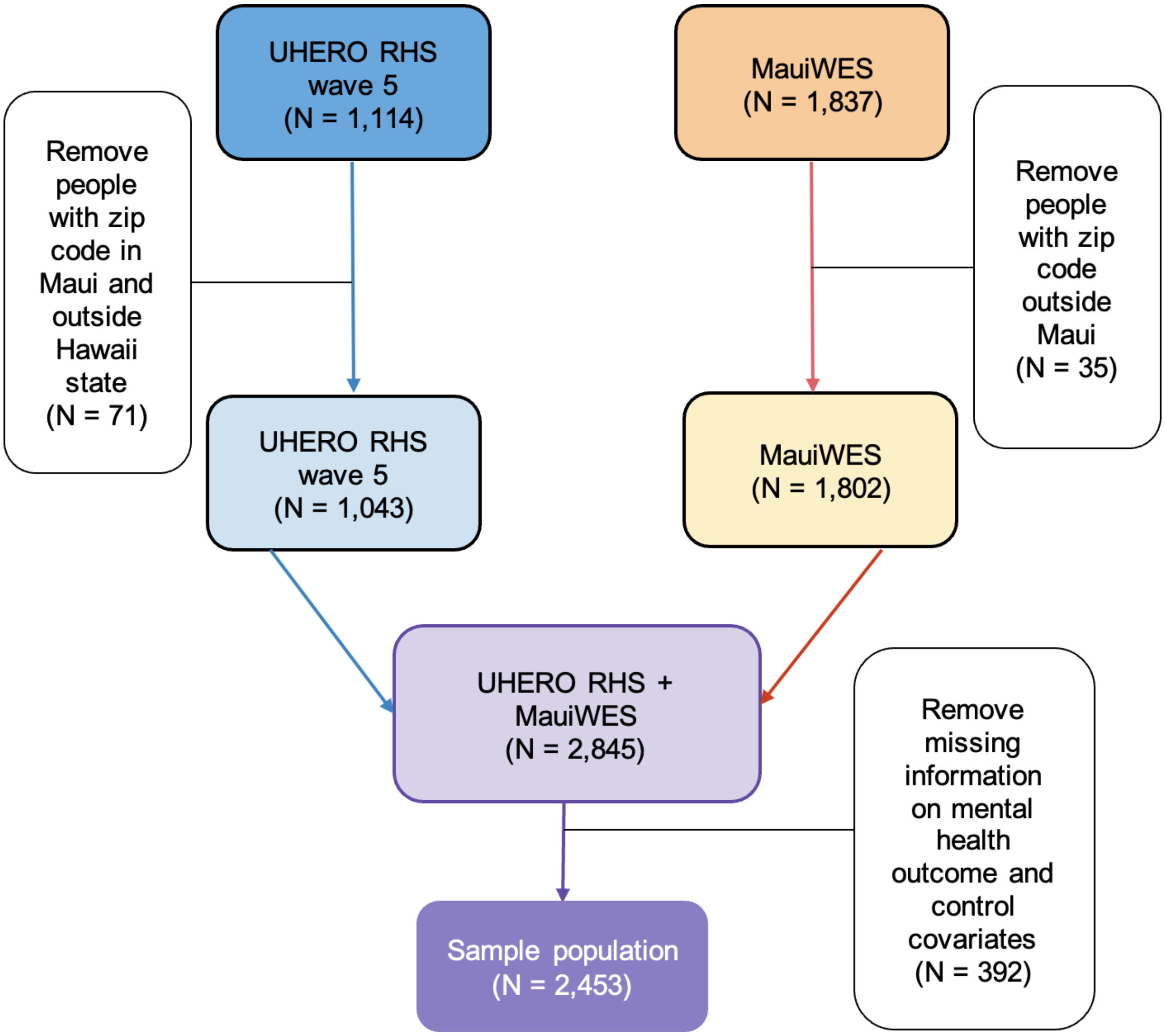

© 2026 Juarez R et al. *JAMA Psychiatry*.

eFigure 2. Monthly Recruitment Distribution for the Maui Wildfire Exposure Study, January 2024-February 2025

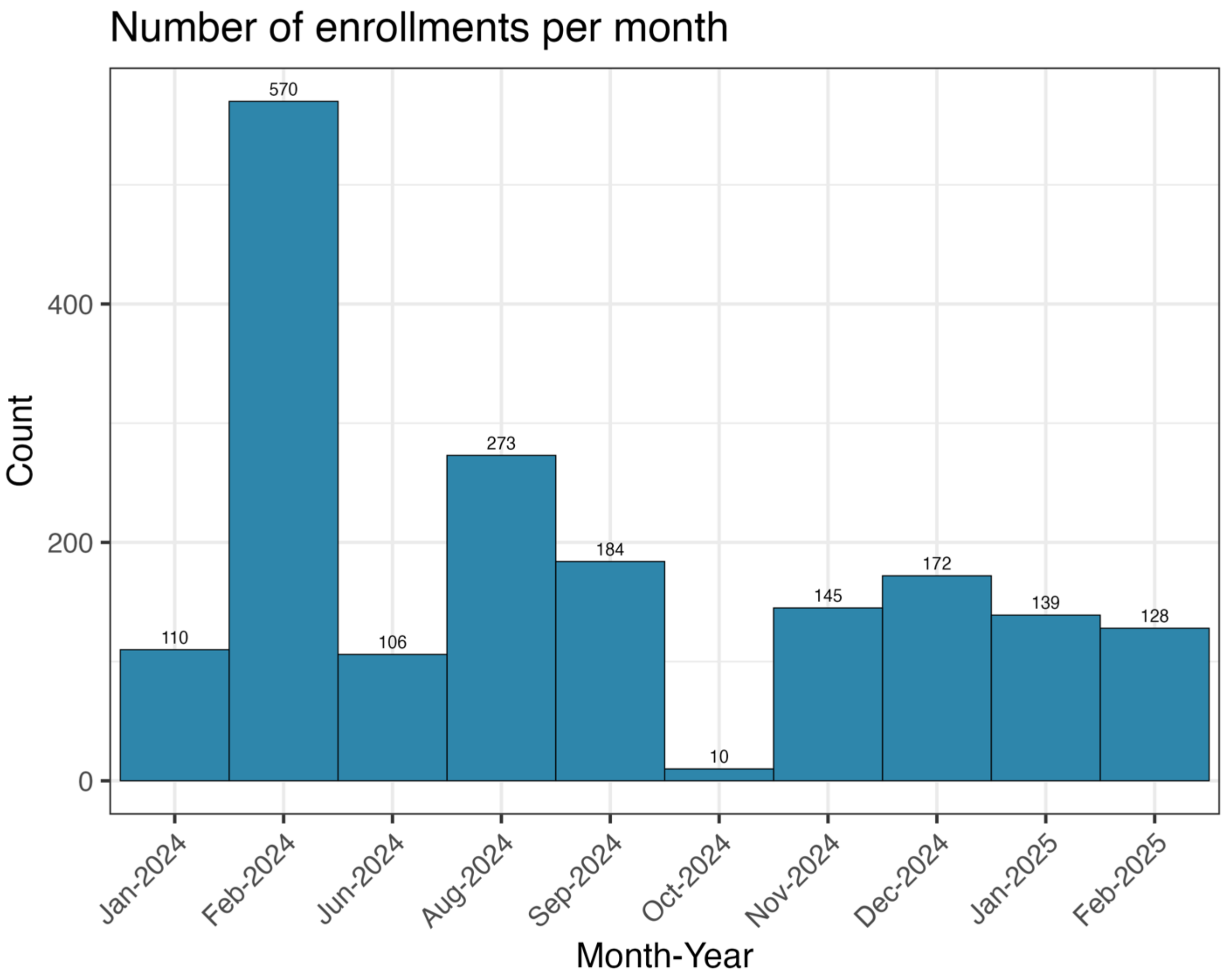

This figure shows monthly enrollments in the MauiWES cohort between January 2024 and February 2025. The median date of enrollment was August 2024, one year after the wildfires.

© 2026 Juarez R et al. *JAMA Psychiatry*.

Descriptive Statistics and Covariate Balance

eFigure 3. Covariate Balance Before and After Propensity Score Weighting

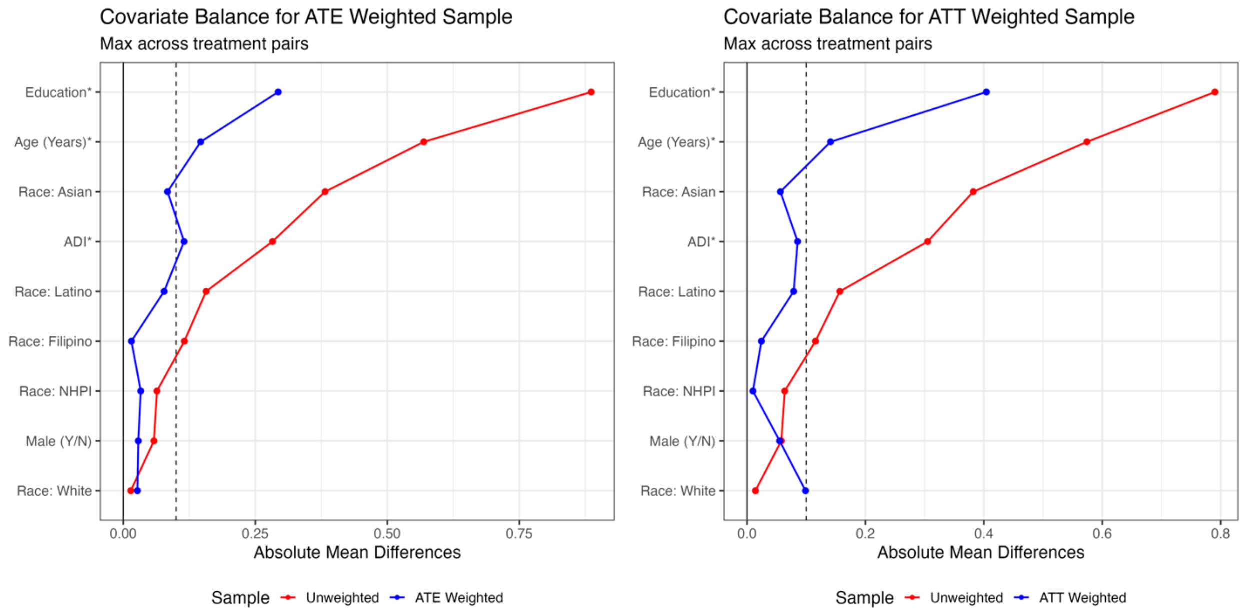

This figure shows the distribution of absolute standardized mean differences (SMDs) for nine pre-treatment covariates—including age, education, race/ethnicity (White, Filipino, Hispanic or Latino, NHPI, and Asian), gender, and average deprivation index—before and after propensity score weighting. Red lines indicate SMDs in the unweighted sample; blue lines represent values after weighting. Two weighting approaches are presented: (1) ATE–weighted sample and (2) ATT–weighted sample (with the burn zone group unweighted). Covariate balance was substantially improved following weighting, with most SMDs falling below the conventional threshold of 0.1 (dashed vertical line). Exceptions were education and age in the ATE-weighted sample and the ATT-weighted sample, which slightly exceeded the threshold.

© 2026 Juarez R et al. *JAMA Psychiatry*.

eFigure 4. Distribution of Mental Health Scores by Wildfire Exposure Group

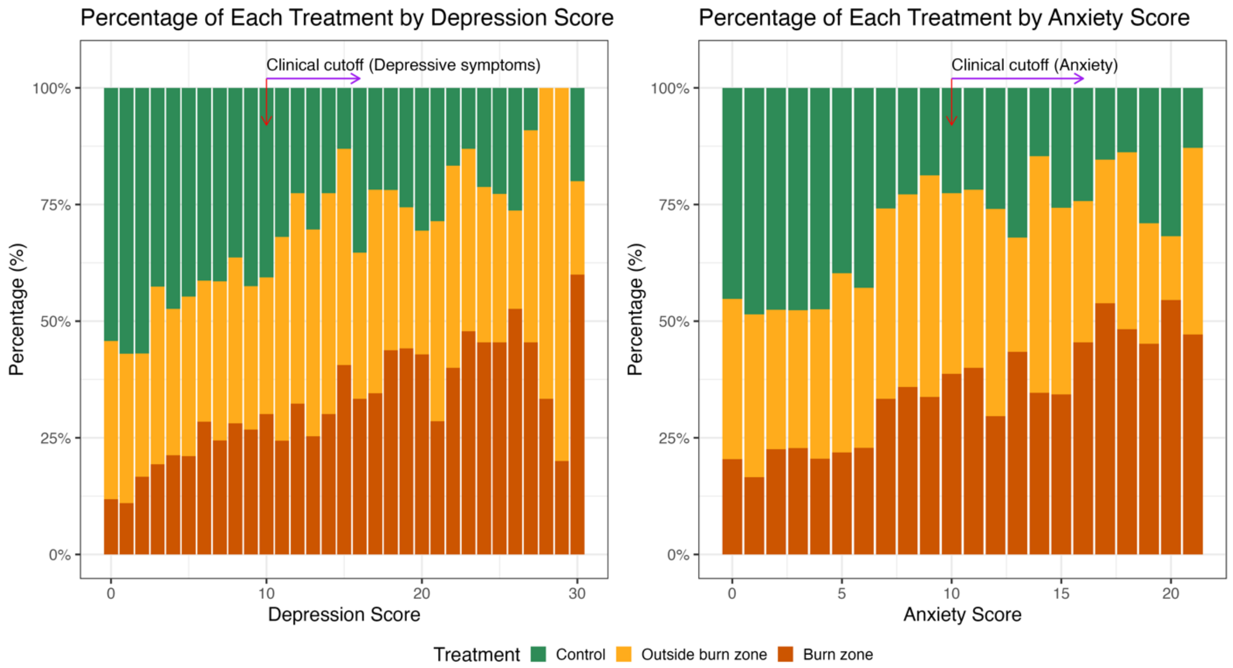

This figure displays the distribution of self-reported mental health scores—depression and anxiety—by exposure group (control, outside burn zone, and burn zone). Depression scores were measured using the 10-item Center for Epidemiologic Studies Depression Scale (CES-D), ranging from 0 to 30. Higher scores indicate greater symptom burden. A score below 10 reflects the absence of depressive symptoms; scores of 10–20 suggest mild-to-moderate symptoms; and scores above 20 reflect a high risk of depression. For analysis, a threshold of ≥10 was used to classify individuals as exhibiting depressive symptoms. Anxiety scores were assessed via the 7-item Generalized Anxiety Disorder scale (GAD-7), ranging from 0 to 21. A score below 10 suggests minimal or mild anxiety, while scores ≥10 indicate moderate-to-severe anxiety. This cutoff was used to define clinically significant anxiety. Across both scales, participants residing in the burn zone reported higher scores and a greater percentage of individuals in the clinically elevated range, consistent with increased mental health burden among those with direct wildfire exposure.

© 2026 Juarez R et al. *JAMA Psychiatry*.

eFigure 5. Geographic Variation in Mental Health Scores by ZIP Code

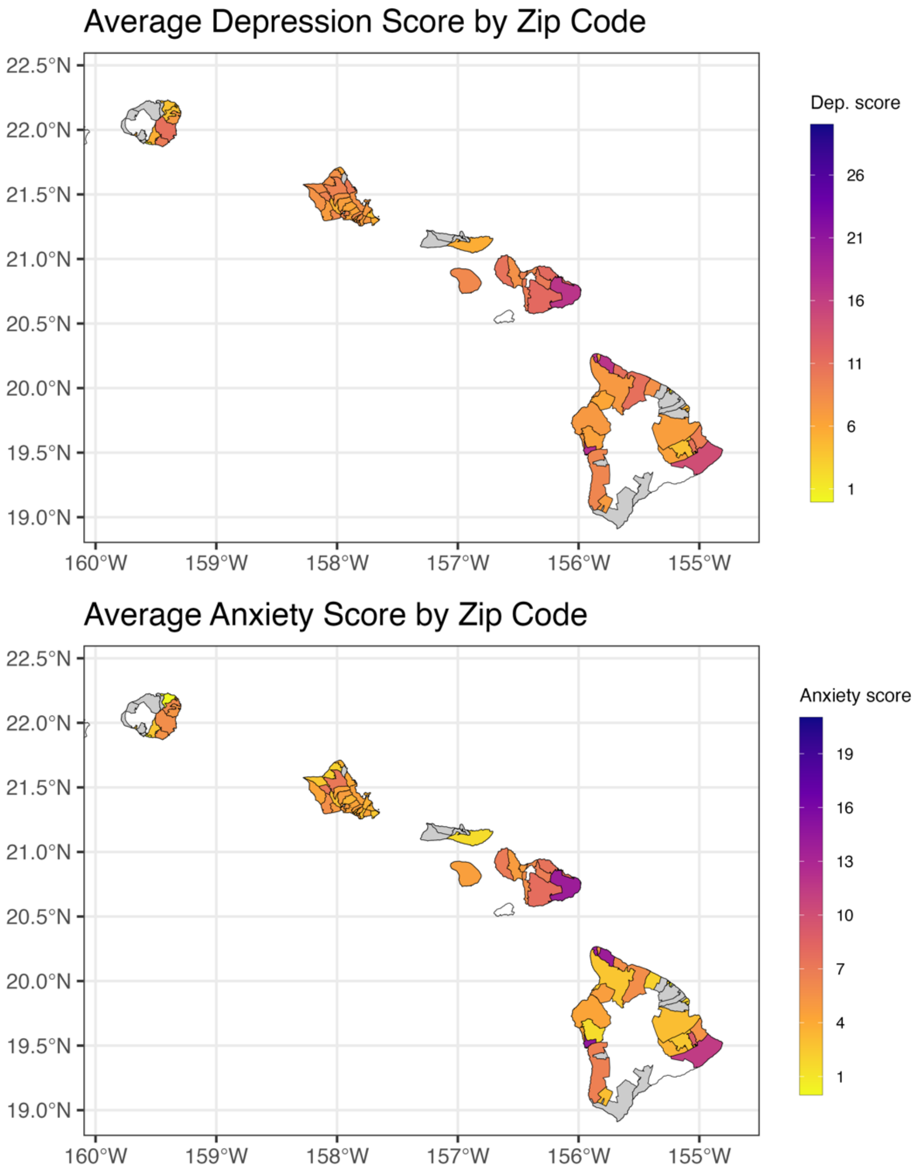

This choropleth map illustrates the geographic distribution of average mental health scores—depression and anxiety—by ZIP code across the state of Hawaii. Scores were derived from individual-level responses in the Maui Wildfire Exposure Study (MauiWES) and the fifth wave of the UHERO Rapid Health Survey (UHERO-RHS). ZIP codes are shaded according to mean mental health scores, with darker colors indicating higher average levels of psychological distress. ZIP codes with no available data are displayed in gray. Blank regions represent mountainous or unpopulated areas not assigned ZIP code polygons. Higher average scores were concentrated in Maui County, particularly in areas affected by the 2023 wildfires, suggesting a geographic clustering of elevated mental health risk. In contrast, lighter shading was observed in ZIP codes within Honolulu, Hawaii, and Kauai counties.

© 2026 Juarez R et al. *JAMA Psychiatry*.

eFigure 6. Geographic Variation in Area Deprivation Index by Census Block Group in 2020

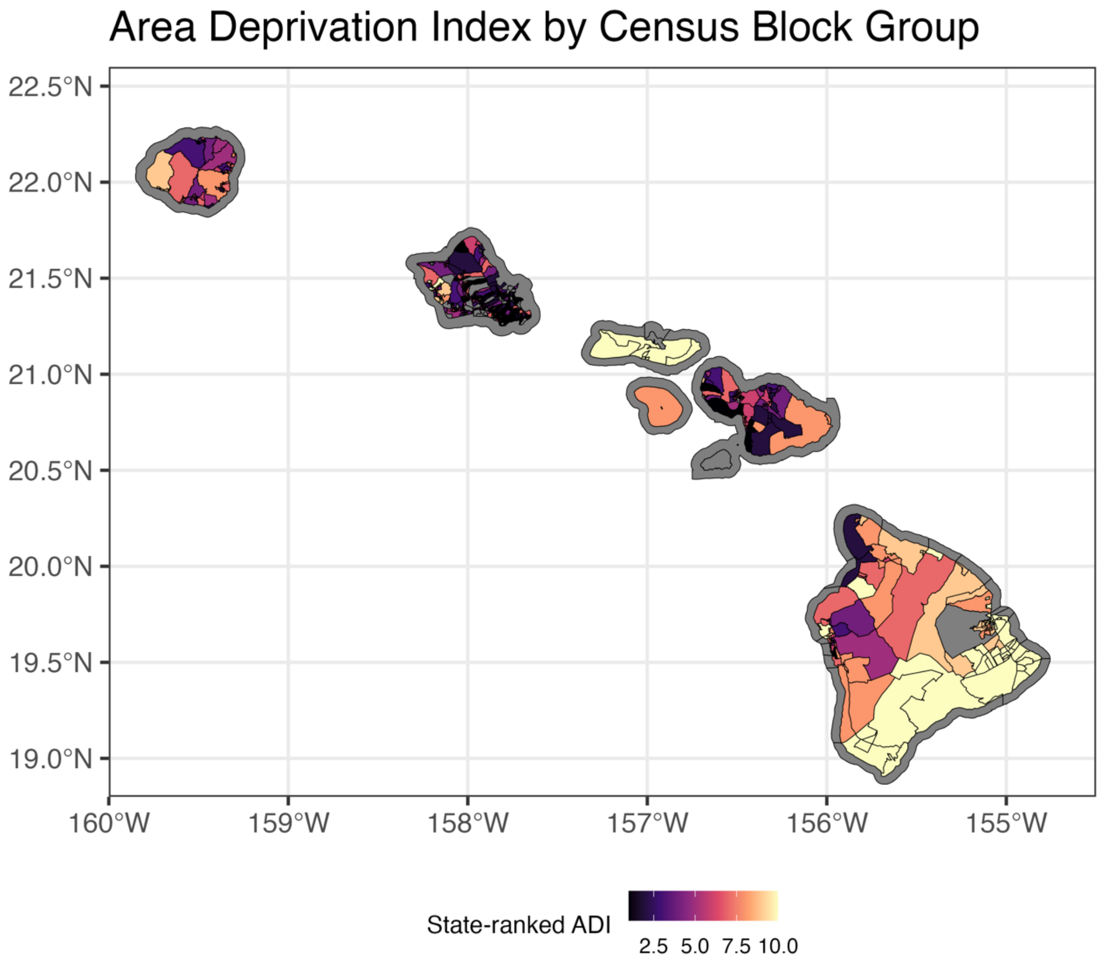

This choropleth map illustrates the geographic distribution of average area deprivation index by census block group across the state of Hawaii. State-ranked ADI score was between 0 and 10 that is publicly available in the University of Wisconsin's Neighborhood Atlas website https://www.neighborhoodatlas.medicine.wisc.edu. Higher score indicates the more disadvantaged socioeconomic area. The graph depicts highly variant across the state. Lower scores were concentrated in Honolulu and Maui counties. In contrast, brighter colors were observed in Hawaii and Molokai counties.

© 2026 Juarez R et al. *JAMA Psychiatry*.

Primary Regression Models

eTable 1. Association Between Wildfire Exposure and Mental Health Outcomes Using Propensity Score–Weighted Models

	Risk Ratio (RR, 95%CI)						Odds Ratio (OR, 95% CI)					
	Depression		Anxiety		Suicidal ideation		Depression		Anxiety		Suicidal ideation	
Burn zone	1.93*** [1.64 ; 2.26]	1.53*** [1.20 ; 1.94]	2.63*** [2.02 ; 3.41]	1.67** [1.14 ; 2.45]	4.18*** [1.99 ; 8.75]	2.15 [0.72 ; 6.44]	2.99*** [2.31 ; 3.89]	1.96** [1.29 ; 2.99]	3.34*** [2.43 ; 4.58]	1.88** [1.17 ; 3.04]	4.31*** [2.03 ; 9.16]	2.17 [0.70 ; 6.75]
Outside burn zone	1.55*** [1.30 ; 1.86]	1.43*** [1.19 ; 1.73]	1.66*** [1.26 ; 2.20]	1.41* [1.05 ; 1.88]	3.34** [1.59 ; 7.04]	2.65* [1.21 ; 5.77]	1.97*** [1.49 ; 2.60]	1.73*** [1.29 ; 2.31]	1.82*** [1.31 ; 2.53]	1.48* [1.05 ; 2.09]	3.42*** [1.60 ; 7.29]	2.69* [1.22 ; 5.96]
Retired		0.64*** [0.53 ; 0.78]		0.44*** [0.31 ; 0.62]		0.41+ [0.15 ; 1.14]		0.43*** [0.30 ; 0.59]		0.34*** [0.23 ; 0.52]		0.39+ [0.14 ; 1.13]
Employed		0.72*** [0.64 ; 0.82]		0.63*** [0.52 ; 0.78]		0.45** [0.26 ; 0.78]		0.51*** [0.40 ; 0.66]		0.53*** [0.39 ; 0.70]		0.44** [0.25 ; 0.77]
House relocation		1.20+ [0.98 ; 1.46]		1.45* [1.07 ; 1.96]		1.69 [0.71 ; 4.02]		1.42+ [0.97 ; 2.09]		1.64* [1.10 ; 2.43]		1.73 [0.70 ; 4.27]

This table presents risks ratios and odds ratios estimating the association between wildfire exposure and mental health outcomes—depression, anxiety, and suicidal ideation—using multi-arm propensity score–weighted logistic regression models. Two models are shown for each outcome: **Model 1** includes wildfire exposure. **Model 2** further adjusts for post-disaster factors, including employment status and housing displacement. Analyses were conducted using inverse probability of treatment weighting for the average treatment effect, yielding an effective sample size of 1,351.53 individuals: 508.65 in the control group, 492.69 in the burn zone group, and 350.19 in the outside burn zone group. Risk ratios or Odds ratios greater than 1 indicate higher odds of adverse mental health outcomes associated with wildfire exposure; values less than 1 suggest protective associations. Ninety-five percent confidence intervals are shown in brackets. Statistical significance is indicated as follows: +p < 0.1, *p < 0.05, **p < 0.01, ***p < 0.001.

© 2026 Juarez R et al. *JAMA Psychiatry*.

Mediation Analysis

eTable 2. Mediation of Wildfire Exposure Effects on Mental Health Outcomes via Housing Relocation and Employment Disruption

Exposure	Outcome	Total Effect (95% CI)	Direct Effect (95% CI)	Indirect via Housing (95% CI)	Indirect via Income Decrease (95% CI)	Mediated (%)
Burn zone	Depression	0.258*** [0.198 ; 0.317]	0.099+ [-0.003 ; 0.201]	0.08* [0.005 ; 0.156]	0.079*** [0.036 ; 0.121]	61.62%
	Anxiety	0.188*** [0.14 ; 0.236]	0.042 -0.034 ; 0.117]	0.08** [0.025 ; 0.135]	0.066*** [0.035 ; 0.098]	77.66%
	Suicidal Ideation	0.031*** [0.014 ; 0.047]	-0.001 [-0.033 ; 0.031]	0.017 [-0.008 ; 0.041]	0.015* [0.003 ; 0.027]	103.23%
Outside burn zone	Depression	0.154*** [0.089 ; 0.218]	0.069+ [-0.001 ; 0.139]	0.023* [0.003 ; 0.042]	0.062*** [0.03 ; 0.094]	54.54%
	Anxiety	0.077*** [0.034 ; 0.119]	0.002 [-0.046 ; 0.050]	0.022** [0.007 ; 0.037]	0.052*** [0.029 ; 0.076]	96.1%
	Suicidal Ideation	0.023** [0.009 ; 0.037]	0.006 [-0.010 ; 0.022]	0.005 [-0.002 ; 0.012]	0.012* [0.003 ; 0.021]	73.91%

This table presents results from structural equation models evaluating the direct and indirect effects of wildfire exposure on mental health outcomes—depression, anxiety, and suicidal ideation—mediated by housing displacement and employment disruption, measured by income decrease. Analyses were conducted using the lavaan.survey package and the inverse probability of treatment–weighted sample for the average treatment effect. The total effect of wildfire exposure is decomposed into direct effects (not mediated) and indirect effects transmitted through post-disaster housing relocation and income decrease. For suicidal ideation among individuals in the burn zone, the estimated direct effect was negative, resulting in a proportion mediated exceeding 100%, which suggests suppression or inconsistent mediation. Ninety-five percent confidence intervals are provided in brackets. Statistical significance is indicated as follows: + $p < 0.1$, *$p < 0.05$, **$p < 0.01$, ***$p < 0.001$.

© 2026 Juarez R et al. *JAMA Psychiatry*.

Sensitivity Analyses

eFigure 7. Sensitivity Analysis: Effects of Wildfire Exposure on Mental Health Using ATT Weighting

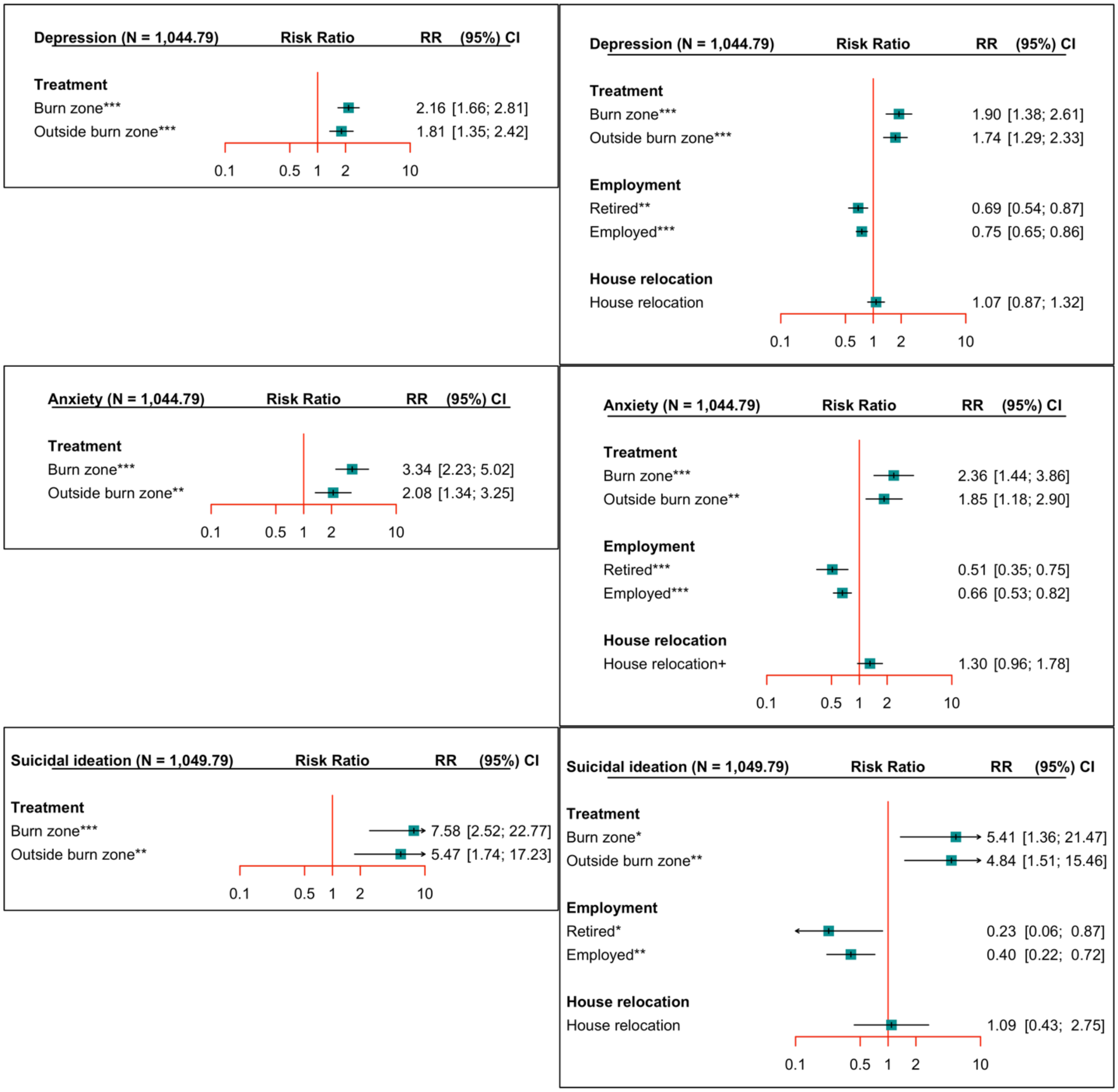

These graphs display risk ratios with 95% confidence intervals for the associations between wildfire exposure and mental health outcomes—depression, anxiety, and suicidal ideation—based on average treatment effect on the treated weighting. Each outcome is modeled using two specifications: **Model 1** includes wildfire exposure. **Model 2** adds employment status and housing relocation as post-disaster structural variables. The ATT-weighted analytic sample includes 1,049.79 individuals: 150.46 in the control group, 658 in the burn zone, and 236.33 outside the burn zone. Matching was performed on across age, gender, education, race/ethnicity (White, Filipino, Hispanic or Latino, NHPI, and Asian), and area deprivation index. Risk ratios are plotted on a logarithmic scale; a red vertical line at RR = 1 indicates the

© 2026 Juarez R et al. *JAMA Psychiatry*.

null. RR >1 indicates higher risk of adverse mental health symptoms associated with wildfire exposure. Statistical significance levels are indicated as follows: + $p < 0.1$, *$p < 0.05$, **$p < 0.01$, ***$p < 0.001$.

© 2026 Juarez R et al. *JAMA Psychiatry*.

eFigure 8. Distribution of Wildfire Exposure Propensity Scores by Treatment Group

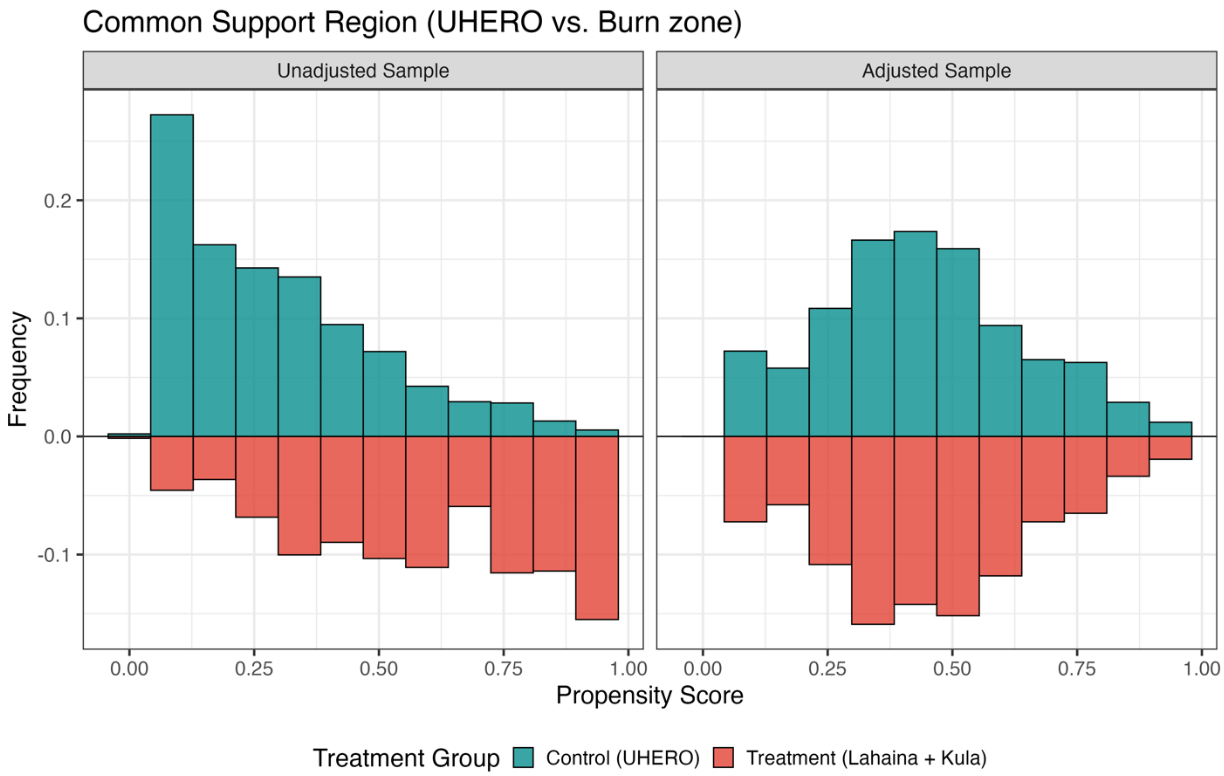

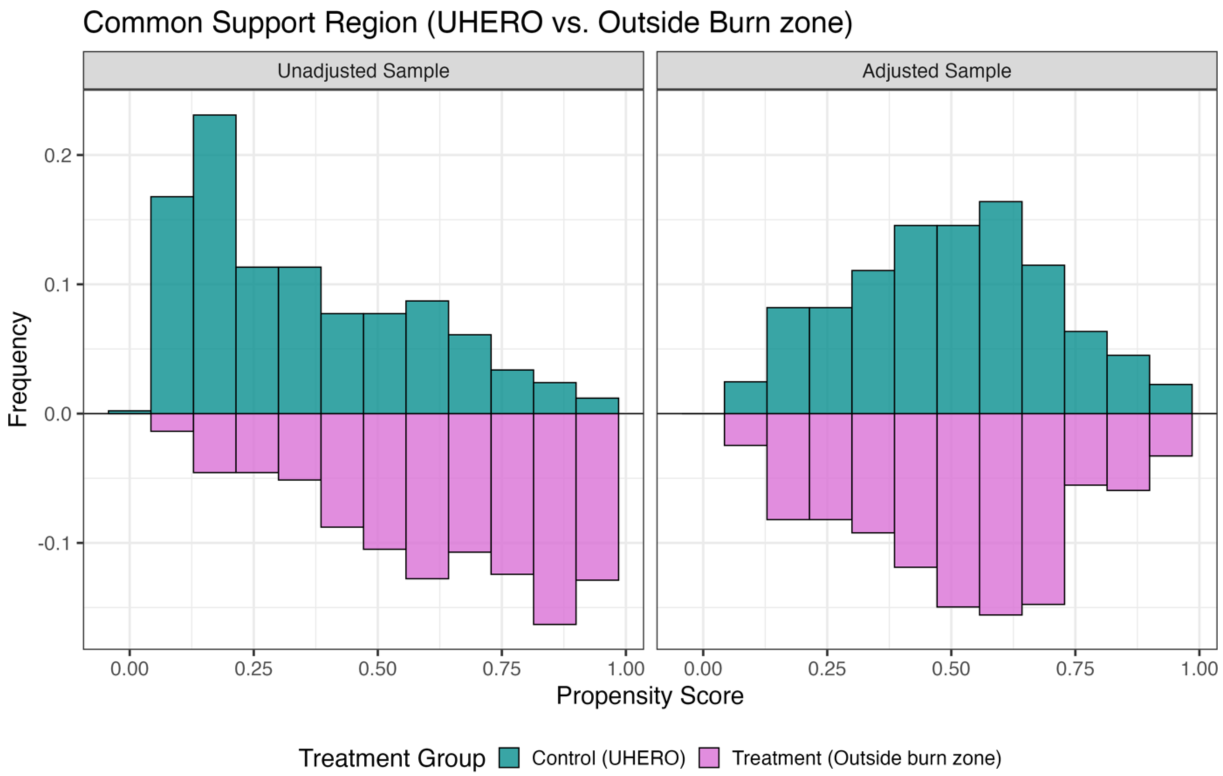

© 2026 Juarez R et al. *JAMA Psychiatry*.

This figure displays the distribution of propensity scores representing the predicted probability of wildfire exposure across three treatment groups: the unexposed UHERO Rapid Health Survey (control group), the MauiWES burn zone group, and the MauiWES outside burn zone group. Propensity scores were derived using pairwise nearest-neighbor matching, within 0.1 caliper to estimate the likelihood of wildfire exposure based on baseline demographic covariates, including age, gender, education, race/ethnicity (White, Filipino, Hispanic or Latino, NHPI, and Asian), and area deprivation index. Distributions show clear separation between groups: individuals in the UHERO control group exhibit significantly lower probabilities of wildfire exposure compared with those in both wildfire-affected groups. Among the MauiWES sample, individuals residing within the burn zone have slightly higher exposure probabilities than those residing outside the burn zone. These patterns affirm the distinct exposure gradients across cohorts and justify the application of propensity score-based methods to adjust for confounding in outcome models. The common support region is presented in the overlapped area, where the meaningful comparison between treatment and control group is analyzed.

© 2026 Juarez R et al. *JAMA Psychiatry*.

eTable 3. Covariate Balance by Treatment Group in Unweighted and ATT-Weighted Samples

	Overall (N = 2,453)	Unweighted sample			Weighted sample		
		Burn zone (N = 658)	Outside burn zone (N = 877)	UHERO (N = 918)	Burn zone (N = 658)	Outside burn zone (N = 236.33)	UHERO (N = 150.46)
**Outcomes**							
**Depression**							
No problem	1,432 (58.38%)	303 (46.05%)	470 (53.59%)	659 (71.79%)	303 (46.05%)	129 (54.79%)	113 (75.01%)
Depressive/ Highly depressive symptom	1,021 (41.62%)	355 (53.95%)	407 (46.41%)	259 (28.21%)	355 (53.95%)	107 (45.21%)	38 (24.99%)
**Anxiety**							
Minimal anxiety/ Mild anxiety	1,954 (79.66%)	455 (69.15%)	686 (78.22%)	813 (88.56%)	455 (69.15%)	191 (80.77%)	137 (90.77%)
Moderate anxiety/ Severe anxiety	499 (20.34%)	203 (30.85%)	191 (21.78%)	105 (11.44%)	203 (30.85%)	45 (19.23%)	14 (9.23%)
**Suicidal ideation**							
No	2,380 (97.02%)	629 (95.59%)	845 (96.35%)	906 (98.69%)	629 (95.59%)	229 (96.82%)	150 (99.42%)
Yes	73 (2.98%)	29 (4.41%)	32 (3.65%)	12 (1.31%)	29 (4.41%)	8 (3.18%)	1 (0.58%)
**Covariates**							
**Age**	50.80 (16.29)	48.89 (15.64)	46.91 (15.51)	55.88 (16.16)	48.89 (15.64)	48.19 (14.75)	50.39 (14.48)
**Gender**							
Female	1,502 (61.23%)	379 (57.60%)	556 (63.40%)	567 (61.76%)	379 (57.6%)	149 (63.11%)	90 (60.14%)
Male	951 (38.77%)	279 (42.40%)	321 (36.60%)	351 (38.24%)	279 (42.4%)	87 (36.89%)	60 (39.86%)
**Education**							
Some schooling, no high school diploma	207 (8.44%)	104 (15.81%)	94 (10.72%)	9 (0.98%)	104 (15.81%)	34 (14.54%)	7 (4.4%)
High school diploma	505 (20.59%)	179 (27.20%)	236 (26.91%)	90 (9.80%)	179 (27.2%)	58 (24.69%)	31 (20.85%)

© 2026 Juarez R et al. *JAMA Psychiatry*.

Some college level/Technical/Vocational degree	713 (29.07%)	196 (29.79%)	269 (30.67%)	248 (27.02%)	196 (29.79%)	75 (31.91%)	52 (34.73%)
Bachelor's degree or higher	1,028 (41.91%)	179 (27.20%)	278 (31.70%)	571 (62.20%)	179 (27.2%)	68 (28.87%)	60 (40.02%)
**Race**							
White							
No	1,439 (58.66%)	392 (59.57%)	510 (58.15%)	537 (58.50%)	392 (59.57%)	137 (57.95%)	75 (49.69%)
Yes	1,014 (41.34%)	266 (40.43%)	367 (41.85%)	381 (41.50%)	266 (40.43%)	99 (42.05%)	76 (50.31%)
Filipino							
No	1,920 (78.27%)	508 (77.20%)	638 (72.75%)	774 (84.31%)	508 (77.2%)	177 (74.77%)	115 (76.52%)
Yes	533 (21.73%)	150 (22.80%)	239 (27.25%)	144 (15.69%)	150 (22.8%)	60 (25.23%)	35 (23.48%)
Hispanic/ Latino							
No	2,164 (88.22%)	529 (80.40%)	753 (85.86%)	882 (96.08%)	364 (80.4%)	193.33 (81.81%)	132.46 (88.04%)
Yes	289 (11.78%)	129 (19.60%)	124 (14.14%)	36 (3.92%)	129 (19.6%)	43 (18.29%)	18 (11.96%)
NHPI							
No	1,923 (78.39%)	528 (80.24%)	653 (74.46%)	742 (80.83%)	528 (80.24%)	191 (81.02%)	120 (80.02%)
Yes	530 (21.61%)	130 (19.76%)	224 (25.54%)	176 (19.17%)	130 (19.76%)	45 (18.98%)	30 (19.98%)
Asian							
No	1,670 (68.08%)	539 (81.91%)	724 (82.55%)	407 (44.34%)	539 (81.91%)	196 (82.75%)	116 (77.11%)
Yes	783 (31.92%)	119 (18.09%)	153 (17.45%)	511 (55.66%)	119 (18.09%)	41 (17.25%)	34 (22.89%)
Other race							
No	2,292 (93.44%)	601 (91.34%)	805 (91.79%)	886 (96.51%)	601 (91.34%)	218 (92.38%)	145 (96.54%)
Yes	161 (6.56%)	57 (8.66%)	72 (8.21%)	32 (3.49%)	57 (8.66%)	18 (7.62%)	5 (3.46%)
House relocation							
No	1,652 (67.35%)	89 (13.53%)	647 (73.77%)	916 (99.78%)	89 (13.53%)	182 (76.99%)	150 (99.96%)

© 2026 Juarez R et al. *JAMA Psychiatry*.

Yes	801 (32.65%)	569 (86.47%)	230 (26.23%)	2 (0.22%)	569 (86.47%)	54 (23.01%)	0 (0.04%)
**Employment status**							
Employed	1,488 (60.66%)	354 (53.80%)	597 (68.07%)	537 (58.50%)	354 (53.8%)	170 (71.76%)	97 (64.59%)
Retired	454 (18.51%)	77 (11.70%)	99 (11.29%)	278 (30.28%)	77 (11.7%)	21 (9.03%)	31 (20.82%)
Unemployed	511 (20.83%)	227 (34.50%)	181 (20.64%)	103 (11.22%)	227 (34.5%)	45 (19.22%)	22 (14.59%)
Area Deprivation Index (ADI)	5.20 (2.67)	5.57 (2.43)	4.83 (2.35)	5.29 (3.05)	5.56 (2.43)	5.36 (2.09)	5.36 (2.51)

This table presents the mean (SD) values for continuous variables and n (%) for categorical variable of key baseline characteristics across wildfire exposure groups—control (UHERO), burn zone, and outside burn zone—before and after applying Average Treatment effect on the Treated weighting. ATT weighting was implemented using a generalized boosted model to enhance covariate comparability and estimate the effect of wildfire exposure among individuals most likely to have been directly impacted by the disaster. The ATT approach reweights individuals in the control and outside burn zone groups to resemble those residing within the burn zone. The effective sample size after ATT weighting includes 1,044.79 individuals: 150.46 in the control group, 658 in the burn zone group, and 236.33 in the outside burn zone group. Matching was based on pre-treatment demographic covariates, including age, gender, education, race/ethnicity (White, Filipino, Hispanic or Latino, NHPI, and Asian), and area deprivation index.

© 2026 Juarez R et al. *JAMA Psychiatry*.

eTable 4. Covariate Balance Across Treatment Groups Using Pairwise Propensity Score Matching

	Burn zone vs. UHERO						Outside burn zone vs. UHERO					
	All data			Matched data (caliper = 0.1)			All data			Matched data (caliper = 0.1)		
	Treat	Control	Diff. in mean	Treat	Control	Diff. in mean	Treat	Control	Diff. in mean	Treat	Control	Diff. in mean
	N = 658	N = 918	(T - C)	N = 415	N = 415	(T - C)	N = 877	N = 918	(T - C)	N = 488	N = 488	(T - C)
**Outcomes**												
Depression	0.539	0.282	0.257***	0.552	0.27	0.282***	0.464	0.282	0.182***	0.451	0.279	0.172***
Anxiety	0.308	0.114	0.194***	0.337	0.118	0.219***	0.218	0.114	0.103***	0.197	0.127	0.07**
Suicidal ideation	0.044	0.0131	0.031***	0.043	0.019	0.024*	0.036	0.013	0.023**	0.037	0.014	0.023*
**Independent variables**												
Employed	0.537	0.584	-0.047+	0.551	0.645	-0.094**	0.681	0.585	0.096***	0.697	0.652	0.045
Unemployed	0.345	0.112	0.233***	0.298	0.134	0.164***	0.206	0.112	0.094***	0.16	0.154	0.006
Retired	0.117	0.303	-0.186***	0.149	0.219	-0.07**	0.113	0.303	-0.19***	0.143	0.195	-0.051*
House relocation	0.865	0.002	0.863***	0.843	0	0.843***	0.262	0.002	0.26***	0.248	0	0.248***
Age	48.892	55.884	-6.992***	51.549	51.648	-0.099	46.908	55.885	-8.977***	49.629	50.725	-1.096
Male	0.424	0.382	0.042+	0.375	0.364	0.011	0.366	0.382	-0.016	0.322	0.35	-0.029
Education	2.683	3.504	-0.821***	3.144	3.205	-0.061	2.834	3.504	-0.671***	3.25	3.291	-0.041
Race												
White	0.404	0.415	-0.011	0.504	0.528	-0.024	0.418	0.415	0.003	0.496	0.539	-0.043
Filipino	0.227	0.156	0.071***	0.241	0.222	0.019	0.273	0.157	0.116***	0.262	0.23	0.033
Latino	0.196	0.0392	0.157***	0.062	0.077	-0.015	0.141	0.039	0.102***	0.076	0.066	0.01
NHPI	0.197	0.1917	0.005	0.207	0.21	-0.002	0.255	0.192	0.064**	0.23	0.227	0.002
Non-Filipino Asian	0.181	0.5567	-0.376***	0.272	0.27	0.002	0.174	0.557	-0.382***	0.285	0.281	0.004
Other races	0.087	0.0348	0.052***	0.094	0.041	0.053**	0.082	0.035	0.047***	0.082	0.035	0.047**
Area Deprivation Index	5.568	5.293	0.275*	5.448	5.533	-0.084	4.826	5.293	-0.467***	5.049	5.135	-0.086

This table presents mean values for key covariates across treatment groups after applying pairwise nearest-neighbor propensity score matching with a caliper of 0.1. Matching was conducted on pre-treatment demographic variables, including age, gender, education, race/ethnicity (White,

© 2026 Juarez R et al. *JAMA Psychiatry*.

Filipino, Hispanic or Latino, NHPI, and Asian), and area deprivation index. Each individual in the wildfire-exposed groups was matched to a counterpart in the control group with a similar likelihood of exposure, ensuring comparability. Two separate matched comparisons are reported: (1) burn zone versus UHERO control (n = 830; 415 per group), and (2) outside burn zone versus UHERO control (n = 976; 488 per group). Figure E3 illustrates the region of common support across matched groups. Post-matching, covariate distributions were well-balanced between treatment and control samples. Statistical significance of group differences is indicated by $+p < 0.1$, $*p < 0.05$, $**p < 0.01$, $***p < 0.001$.

© 2026 Juarez R et al. *JAMA Psychiatry*.

Additional Robustness Checks

eTable 5. Association Between Wildfire Exposure and Mental Health Outcomes Using ATT Weighting

	Risk Ratio (RR, 95%CI)						Odds Ratio (OR, 95% CI)					
	Depression		Anxiety		Suicidal ideation		Depression		Anxiety		Suicidal ideation	
Burn zone	2.16*** [1.66 ; 2.81]	1.90*** [1.38 ; 2.61]	3.34*** [2.23 ; 5.02]	2.36*** [1.44 ; 3.86]	7.58*** [2.52 ; 22.77]	5.41* [1.36 ; 21.47]	3.52*** [2.42 ; 5.1]	2.78*** [1.66 ; 4.64]	4.39*** [2.77 ; 6.96]	2.81*** [1.57 ; 5.03]	7.88*** [2.59 ; 23.95]	5.59* [1.36 ; 23.04]
Outside burn zone	1.81*** [1.35 ; 2.42]	1.74*** [1.29 ; 2.33]	2.08** [1.34 ; 3.25]	1.85** [1.18 ; 2.90]	5.47** [1.74 ; 17.23]	4.84** [1.51 ; 15.46]	2.48*** [1.62 ; 3.79]	2.32*** [1.50 ; 3.58]	2.34*** [1.41 ; 3.88]	2.03** [1.21 ; 3.38]	5.62** [1.76 ; 17.91]	4.98** [1.53 ; 16.19]
Retired		0.69** [0.54 ; 0.87]		0.51*** [0.35 ; 0.75]		0.23* [0.06 ; 0.87]		0.48*** [0.31 ; 0.72]		0.41*** [0.25 ; 0.66]		0.22* [0.06 ; 0.85]
Employed		0.75*** [0.65 ; 0.86]		0.66*** [0.53 ; 0.82]		0.39** [0.22 ; 0.72]		0.55*** [0.41 ; 0.74]		0.55*** [0.40 ; 0.76]		0.38** [0.20 ; 0.71]
House relocation		1.07 [0.87 ; 1.32]		1.30+ [0.96 ; 1.78]		1.09 [0.43 ; 2.75]		1.15 [0.76 ; 1.74]		1.43+ [0.95 ; 2.14]		1.09 [0.41 ; 2.88]

This table reports the estimated effects of wildfire exposure on three mental health outcomes—depression, anxiety, and suicidal ideation—based on risk ratios and odds ratios (ORs) derived from average treatment effect on the treated multi-arm propensity score models. Two model specifications are shown for each outcome: **Model 1** includes wildfire exposure; **Model 2** adds employment status and housing relocation as additional covariates. The ATT-weighted analytic sample includes 1,044.79 individuals: 150.46 in the control group, 658 in the burn zone group, and 236.33 in the outside burn zone group. Matching was performed using generalized boosted models to approximate the counterfactual mental health burden among those most likely to experience wildfire exposure. A RR > 1 or an OR >1 indicates higher risk or odds of adverse mental health outcomes associated with exposure; RR < 1 or OR <1 indicates protective associations. Each cell presents the estimated RR or OR and corresponding 95% confidence interval. Statistical significance is indicated as follows: + $p < 0.1$, *$p < 0.05$, **$p < 0.01$, ***$p < 0.001$.

© 2026 Juarez R et al. *JAMA Psychiatry*.

eTable 6. Effects of Wildfire Exposure on Mental Health Outcomes Using Pairwise Propensity Score Matching

	Risk ratio (95%CI)						Odds ratio (95% CI)					
**Variables**	**Depression**		**Anxiety**		**Suicidal ideation**		**Depression**		**Anxiety**		**Suicidal ideation**	
	**UHERO vs. Burn zone**	**UHERO vs. outside burn zone**	**UHERO vs. Burn zone**	**UHERO vs. outside burn zone**	**UHERO vs. Burn zone**	**UHERO vs. outside burn zone**	**UHERO vs. Burn zone**	**UHERO vs. outside burn zone**	**UHERO vs. Burn zone**	**UHERO vs. outside burn zone**	**UHERO vs. Burn zone**	**UHERO vs. outside burn zone**
Burn zone	1.78*** [1.33 ; 2.38]		2.14** [1.31 ; 3.48]		2.80+ [0.87 ; 9.03]		2.53*** [1.48 ; 4.34]		2.53** [1.32 ; 4.71]		2.96+ [0.76 ; 9.84]	
Outside burn zone		1.46*** [1.21 ; 1.76]		1.35+ [0.98 ; 1.86]		1.94 [0.75 ; 5.03]		1.77*** [1.33 ; 2.37]		1.43+ [0.98 ; 2.09]		1.98 [0.75 ; 5.50]
Retired	0.71** [0.56 ; 0.92]	0.75+ [0.55 ; 1.01]	0.61* [0.41 ; 0.91]	0.35*** [0.19 ; 0.66]	0.3+ [0.09 ; 1.05]	0.42 [0.11 ; 1.65]	0.51** [0.32 ; 0.81]	0.64+ [0.39 ; 1.03]	0.49* [0.28 ; 0.84]	0.30*** [0.14 ; 0.59]	0.28+ [0.06 ; 0.92]	0.41 [0.09 ; 1.48]
Employed	0.75*** [0.64 ; 0.89]	0.96 [0.78 ; 1.19]	0.67** [0.51 ; 0.87]	0.79 [0.57 ; 1.11]	0.31** [0.13 ; 0.71]	0.45+ [0.19 ; 1.048]	0.55*** [0.38 ; 0.79]	0.93 [0.64 ; 1.35]	0.55** [0.37 ; 0.81]	0.74 [0.48 ; 1.17]	0.29** [0.12 ; 0.69]	0.43+ [0.18 ; 1.11]
House relocation	1.10 [0.85 ; 1.43]	1.39** [1.14 ; 1.70]	1.28 [0.83 ; 1.97]	1.43+ [0.99 ; 2.07]	0.56 [0.19 ; 1.66]	2.15 [0.84 ; 5.49]	1.25 [0.73 ; 2.14]	1.94** [1.28 ; 2.96]	1.43 [0.80 ; 2.68]	1.60+ [0.97 ; 2.60]	0.54 [0.18 ; 1.99]	2.24 [0.83 ; 5.90]

This table presents estimated risk ratios or odds ratios for the association between wildfire exposure and mental health outcomes—depression, anxiety, and suicidal ideation—using pairwise propensity score matching. Two matched comparisons are shown: (1) individuals residing in the burn zone matched to individuals in the UHERO control cohort (N = 830; 415 per group), and (2) individuals residing outside the burn zone matched to UHERO controls (N = 976; 488 per group). Matching was performed using nearest-neighbor algorithms with a caliper of 0.1 on demographic covariates including age, gender, education, race/ethnicity (White, Filipino, Hispanic or Latino, and NHPI), and deprivation, ensuring comparability between exposed and control participants. Each outcome was reported in both risk ratios and odds ratios. RRs and ORs reflect the relative likelihood of adverse mental health outcomes in exposed groups compared to matched controls. A RR > 1 or an OR >1 indicates increased risks or odds of psychological distress associated with wildfire exposure; RR < 1 or OR <1 indicates lower risks or odds; RR = 1 or OR = 1 indicates no association. Each cell reports the RR or OR and corresponding 95% confidence interval. Statistical significance is denoted by: +p < 0.1, *p < 0.05, **p < 0.01, ***p < 0.001.

© 2026 Juarez R et al. *JAMA Psychiatry*.

eTable 7. Association Between Wildfire Exposure and Mental Health Outcomes in State Population-Weighted Sample Using Multi-Arm Propensity Score Matching

	RR (95% CI)						OR (95%CI)					
	Depression		Anxiety		Suicidal ideation		Depression		Anxiety		Suicidal ideation	
Burn zone	1.89*** [1.56 ; 2.29]	1.42* [1.06 ; 1.90]	2.55*** [1.87 ; 3.47]	1.65* [1.06 ; 2.58]	5.2*** [2.52 ; 10.71]	1.42* [1.06 ; 1.90]	2.84*** [2.06 ; 3.90]	1.69* [1.02 ; 2.80]	3.15*** [2.17 ; 4.56]	1.83* [1.05 ; 3.17]	2.84*** [2.06 ; 3.90]	2.53+ [0.89 ; 7.16]
Outside burn zone	1.58*** [1.31 ; 1.90]	1.40** [1.14 ; 1.71]	1.76*** [1.28 ; 2.41]	1.43* [1.02 ; 1.80]	4.75*** [2.29 ; 9.87]	1.40** [1.14 ; 1.71]	2.01*** [1.52 ; 2.66]	1.64*** [1.21 ; 2.23]	1.94*** [1.35 ; 2.79]	1.50* [1.02 ; 2.22]	2.01*** [1.52 ; 2.66]	3.56*** [1.64 ; 7.72]
Retired		0.62*** [0.49 ; 0.79]		0.40*** [0.26 ; 0.61]		0.62*** [0.49 ; 0.79]		0.40*** [0.27 ; 0.6]		0.31*** [0.19 ; 0.52]		0.42 [0.15 ; 1.21]
Employed		0.72*** [0.63 ; 0.82]		0.65*** [0.52 ; 0.83]		0.72*** [0.63 ; 0.82]		0.51*** [0.38 ; 0.67]		0.55*** [0.40 ; 0.77]		0.54* [0.31 ; 0.95]
House relocation		1.27* [1.02 ; 1.59]		1.42* [1.01 ; 2.00]		1.27* [1.02 ; 1.59]		1.60* [1.05 ; 2.44]		1.59* [1.02 ; 2.47]		1.98 [0.85 ; 4.62]

This table presents risk ratios and odds ratios estimating the effect of wildfire exposure on depression, anxiety, and suicidal ideation using multi-arm propensity score matching in a sample reweighted to reflect the Hawaii state population distribution by age and gender. The results confirm the robustness of the primary findings. The effective sample size after average treatment effect weighting includes 1,351.53 individuals: 508.65 in the control group, 429.69 in the burn zone group, and 350.19 in the outside burn zone group. Matching was conducted on key demographic variables including age, gender, education, race/ethnicity (White, Filipino, Hispanic or Latino, NHPI, and Asian), and area deprivation index. Risk ratios and Odds ratios are displayed for each mental health outcome. A RR or an OR >1 indicates increased risks or odds of the outcome associated with wildfire exposure; RR < 1 or OR <1 indicates decreased risks or odds; RR = 1 or OR = 1 indicates no association. Brackets contain 95% confidence intervals. Statistical significance is denoted as follows: + $p < 0.1$, *$p < 0.05$, **$p < 0.01$, ***$p < 0.001$.

© 2026 Juarez R et al. *JAMA Psychiatry*.

eTable 8. Association Between Wildfire Exposure and Mental Health Outcomes Using Pre-Wildfire Controls From UHERO-RHS Wave 3

	RR (95% CI)				OR (95% CI)			
	Depression		Suicidal ideation		Depression		Suicidal ideation	
Burn zone	1.56*** [1.33 ; 1.81]	1.32** [1.09; 1.61]	2.48* [1.15 ; 5.36]	1.30 [0.47 ; 3.60]	2.20*** [1.68 ; 2.86]	1.64** [1.14 ; 2.37]	2.55* [1.16 ; 5.61]	1.30 [0.45 ; 3.73]
Outside burn zone	1.28** [1.08 ; 1.52]	1.21* [1.02 ; 1.43]	2.05+ [0.94 ; 4.51]	1.67 [0.76 ; 3.70]	1.50** [1.13 ; 1.98]	1.36* [1.02 ; 1.79]	2.09+ [0.94 ; 4.67]	1.69 [0.75 ; 3.82]
Retired		0.59*** [0.49 ; 0.71]		0.50 [0.19 ; 1.30]		0.33*** [0.23 ; 0.47]		0.48 [0.18 ; 1.30]
Employed		0.64*** [0.57 ; 0.72]		0.43** [0.250 ; 0.74]		0.38*** [0.29 ; 0.50]		0.41** [0.23 ; 0.73]
House relocation		1.07 [0.90 ; 1.27]		1.77 [0.80 ; 3.94]		1.16 [0.81 ; 1.66]		1.82 [0.79 ; 4.23]

This table presents risk ratios and odds ratios estimating the association between wildfire exposure and mental health outcomes—specifically, depression and suicidal ideation—using multi-arm propensity score matching. Because of data availability, two different mental outcomes were measured - depression and suicidal ideation. The control group comprises individuals surveyed in the third wave of the UHERO Rapid Health Survey (UHERO-RHS) conducted in May 2023, prior to the August 2023 Maui wildfires. The average treatment effect weighted sample includes 1340.11 individuals: 474.58 in the control group, 503.13 in the burn zone group, and 362.40 in the outside burn zone group. Matching was performed on demographic covariates including age, gender, education, race/ethnicity (White, Filipino, Hispanic or Latino, NHPI, and Asian), and area deprivation index. Risk ratios and odds ratios represent the likelihood of adverse mental health outcomes associated with wildfire exposure. A RR > 1 or an OR >1 indicates increased risks or odds; RR < 1 or OR < 1 indicates decreased risks or odds; RR = 1 or OR = 1 suggests no association. Brackets indicate 95% confidence intervals. Statistical significance is denoted as: + $p < 0.1$, *$p < .05$, **$p < .01$, ***$p < .001$.

© 2026 Juarez R et al. *JAMA Psychiatry*.

eTable 9. Robustness Check With E-Values for the Association Between Wildfires Exposure and Mental Health Outcomes

Association	Estimates (RR), 95% CI		E-value for the Point Estimates		E-value for the confidence interval	
	Crude model	Adjusted model	Crude model	Adjusted model	Crude model	Adjusted model
/ ***Depression						
Burn zone	1.93 [1.64 ; 2.26]	1.53 [1.20 ; 1.94]	3.26	2.42	2.66	1.70
Outside burn zone	1.55 [1.30 ; 1.86]	1.43 [1.19 ; 1.73]	2.48	2.22	1.92	1.67
Retired		0.64 [0.53 ; 0.78]		2.49		1.89
Employed		0.73 [0.64 ; 0.82]		2.10		1.74
House relocation		1.20 [0.98 ; 1.46]		1.68		1
/ ***Anxiety						
Burn zone	2.63 [2.02 ; 3.41]	1.67 [1.14 ; 2.45]	4.69	2.73	3.46	1.55
Outside burn zone	1.66 [1.26 ; 2.20]	1.41 [1.05 ; 1.88]	2.72	2.16	1.83	1.28
Retired		0.44 [0.31 ; 0.62]		4.02		2.60
Employed		0.63 [0.52 ; 0.78]		2.53		1.90
House relocation		1.45 [1.07 ; 1.96]		2.26		1.34
/ ***Suicidal ideation						
Burn zone	4.18 [1.99 ; 8.75]	2.15 [0.72 ; 6.44]	7.82	3.73	3.40	1
Outside burn zone	3.34 [1.59 ; 7.04]	2.65 [1.21 ; 5.77]	6.14	4.73	2.55	1.72
Retired		0.41 [0.15 ; 1.14]		4.31		1
Employed		0.45 [0.26 ; 0.78]		3.85		1.90
House relocation		1.69		2.77		1

© 2026 Juarez R et al. *JAMA Psychiatry*.

		[0.71 ; 4.02]				

This table presents risk ratios and corresponding E-values assessing sensitivity to unmeasured confounding in the association between wildfire exposure and mental health outcomes—depression, anxiety, and suicidal ideation—using multi-arm propensity score matching in crude and adjusted models. Matching was performed on age, gender, education, race/ethnicity (White, Filipino, Hispanic or Latino, NHPI, and Asian), and area deprivation index. The average treatment effect weighted sample included 1,351.53 individuals (508.65 control, 429.69 burn zone, and 350.19 outside burn zone). The E-value for the point estimate represents the minimum strength of association an unmeasured confounder would need with both exposure and outcome (on the risk ratio scale) to fully explain away the observed association, while the E-value for the confidence interval indicates the strength required to move the interval to include the null (RR=1). For burn-zone exposure, point-estimate E-values exceeded 2.4 for depression and 2.7 for anxiety (CI limits >1.5), suggesting moderate robustness to unmeasured confounding. In contrast, the suicidal ideation association (E-value ≈1) was more sensitive to potential unmeasured confounding.

© 2026 Juarez R et al. *JAMA Psychiatry*.
