# Supplementary material for: Housing Displacement, Employment Disruption, and Mental Health After the 2023 Maui Wildfires
Source: JAMA Psychiatry. 2026 Mar 11;83(6):601–10. doi: 10.1001/jamapsychiatry.2026.0044 (PMC12980362; doi:10.1001/jamapsychiatry.2026.0044)
Supplement: Supplement 2. — Data Sharing Statement [file jamapsychiatry-e260044-s002.pdf]

Data Sharing Statement

Juarez. Housing Displacement, Employment Disruption, and Mental Health After the 2023 Maui Wildfires. *JAMA Psychiatry*. Published March 11, 2026.
doi:10.1001/jamapsychiatry.2026.0044

Data
Data available: Yes
Data types: Deidentified participant data, Data dictionary
How to access data: Data will be available in de-identified form, contingent on approval by the Scientific Advisory Board SAB and Community Advisory Board CAB of MauiWES. Access requests can be directed to the contact author.
When available: With publication

Supporting Documents
Document types: None

Additional Information
Who can access the data: Qualified researchers and government organizations whose proposed use of the data has been reviewed and approved by the SAB and CAB of MauiWES.
Types of analyses: For any scientifically or community purpose.
Mechanisms of data availability: Through a signed data access agreement after approval by the SAB and CAB of MauiWES.
